# Emerging stock market volatility and economic fundamentals: the importance of US uncertainty spillovers, financial and health crises

**DOI:** 10.1007/s10479-021-04042-y

**Published:** 2021-04-21

**Authors:** M. Karanasos, S. Yfanti, J. Hunter

**Affiliations:** 1 Department of Economics and Finance, Brunel University London, Kingston Lane, Uxbridge, UB8 3PH UK; 2 School of Business and Economics, Loughborough University, Epinal Way, Loughborough, LE11 3TU UK

**Keywords:** Economic policy uncertainty, Emerging markets, Financial and health crises, Macro-financial linkages, Realized variance, Uncertainty spillovers, C22, C58, D80, E44, G01, G15

## Abstract

This paper studies the US and global economic fundamentals that exacerbate emerging stock markets volatility and can be considered as systemic risk factors increasing financial stability vulnerabilities. We apply the bivariate HEAVY system of daily and intra-daily volatility equations enriched with powers, leverage, and macro-effects that improve its forecasting accuracy significantly. Our macro-augmented asymmetric power HEAVY model estimates the inflammatory effect of US uncertainty and infectious disease news impact on equities alongside global credit and commodity factors on emerging stock index realized volatility. Our study further demonstrates the power of the economic uncertainty channel, showing that higher US policy uncertainty levels increase the leverage effects and the impact from the common macro-financial proxies on emerging markets’ financial volatility. Lastly, we provide evidence on the crucial role of both financial and health crisis events (the 2008 global financial turmoil and the recent Covid-19 pandemic) in raising markets’ turbulence and amplifying the volatility macro-drivers impact, as well.

**Supplementary Information:**

The online version supplementary material available at 10.1007/s10479-021-04042-y.

## Introduction

A common stylized fact about emerging economies is the high volatility of their stock markets (De Santis 1997; Aggarwal et al. 1999; Xu 1999; Cano-Berlanga and Giménez-Gómez 2018). The liberalization of the emerging world’s financial markets, which attracted a significant amount of capital flows by foreign institutional investors, has been the first step of an integration process with significant economies’ interdependence and asset markets’ synchronization. Given that emerging economies are characterized by critical vulnerabilities to external shocks, they exhibit higher equity market fluctuations than the developed markets and it is worth investigating how US and global common economic forces affect their intra-daily volatility. In general, modeling the volatility of financial returns has crucial implications for asset allocation, risk management practices, and financial stability oversight. Robust modeling and reliable forecasting of the volatility trajectory of financial instruments has been the main task and objective of financial economics applications for business operations, given that volatility constitutes one of the fundamental input variables in estimations and decision processes of any corporation on investing and funding choices. Financial volatility is also closely inspected by policymakers since it is perceived to constitute an early warning crisis signal, entailing critical destabilizing threats for the financial system (see, for example, Kürüm et al. 2018).

This paper applies an extension of the bivariate HEAVY^1^ system, firstly, with asymmetries and power transformations, through the APARCH structure of Ding et al. (1993). Motivated by the widely-recognized merits of the this framework, which considerably improves Bollerslev’s (1986) GARCH process by adding leverage and power effects (see, for example, Karanasos and Kim 2006), we similarly extend the HEAVY system with these two main features to demonstrate its superiority over the benchmark specification introduced by Shephard and Sheppard (2010). The optimal estimation of the power term and the asymmetric response to positive and negative shocks embedded in the time-varying volatility pattern have already proved to be one of the most pivotal innovations in the GARCH family of models (see, for example, Brooks et al. 2000). One of our first findings is that each of the two powered conditional variances is significantly affected by the first lags of both power transformed variables, that is, squared negative returns, and realized variance. Secondly, we extend the asymmetric power specification with macro-effects from US economic policy and financial uncertainty, bond and commodity market global benchmarks, and the infectious disease news impact on US equity market volatility, providing a competing framework of volatility modeling to the well-established practice of financial instruments trading and risk measuring based on economic fundamentals. We apply the macro-augmented model on five emerging stock market indices from two different regions: the Americas and Asia-Pacific. The realized measure receives significant positive impact from all macro-variables included with further improvement of the model’s forecasting performance. Moreover, we examine not only the direct destabilizing effect of the policy uncertainty volatility spillover on stock market realized volatility, by using it as a conditional variance regressor, but also the policy uncertainty level impact on each parameter of the system (indirect impact), showing that higher US policy uncertainty inflates the leverage and the macro-environment’s effects from financial uncertainty, credit conditions, commodity markets, and infectious diseases on the realized measure. Finally, we explore the crisis influence on emerging stock markets and find that both global financial (the 2008 financial turmoil) and health (the Covid-19 outbreak) crisis events magnify the markets turbulence and the volatility macro-drivers effect.

At the beginning of the 2008 global financial crisis, when volatilities increased sharply and persistently with crucial systemic risk externalities, we witnessed a reigniting interest of regulators and academics in meaningful volatility estimates, while, at the same time, practitioners remained alert to improving the relevant volatility frameworks on a day-to-day basis. Financial economics scholars focused on volatility as a potent catalyst of systemic risk build-up, which policymakers tried to limit. We demarcate this study from the extant finance bibliography by applying the extended HEAVY model with asymmetries, power transformations, and macro-effects, a well-defined framework that adequately fits the volatility process and considerably outperforms the traditional econometric approaches for modeling returns [GARCH(1, 1) specifications] and realized volatility (long memory specifications: Autoregressive Fractionally Integrated Moving Average-ARFIMA(1, *d*, 1) and Heterogeneous Autoregressive Realized Variance-HAR-RV). Our framework contributes to two main strands of empirical macro-finance literature: the research on volatility modeling and the macro-financial linkages in emerging economies, with the investigation of the crucial US uncertainty spillover effects and crisis events on emerging financial market stability. Most importantly, we intend to complement existing evidence by bridging the research on the macro-relevance of financial volatility with the high-frequency financial data domain. Filling a notable gap of the academic literature related to the high-frequency macro-financial linkages in emerging economies our novel findings are summarized as follows: (i) higher volatility of US Economic Policy Uncertainty (EPU), elevated US financial uncertainty, tighter credit conditions, increased commodity prices, and stronger infectious disease news impact on US equities, all five economic forces intensify emerging stock markets volatilities, (ii) the economic uncertainty channel (proxied by the US EPU level) further exacerbates asymmetries and macro-ramifications on emerging equities, and (iii) both global crisis events, the subprime crisis and the current pandemic-led crash, boost emerging stock market volatility through the time-varying pattern of the HEAVY’s parameters with the macro-effects included, as well.

In this vein, our analysis focuses on the macro-financial linkages running from the macroeconomy to the financial sector by incorporating important economic fundamentals in emerging equity volatility modeling for a long sample period covering the recent health crisis-driven market crash. We aim at contributing to the existing empirical evidence on the macro-factors driving financial volatility by using daily macro-variables (instead of monthly or quarterly proxies included in the existing literature) and on the effects of the global financial turmoil and the recent pandemic on volatility dynamics. The higher the frequency of economic news incorporated in forecasting the volatility pattern, the more accurately the predicted values will be. Daily volatility forecasts, updated with daily shocks from the continuously evolving macro-environment, offer the necessary tools for market participants closely watching day-to-day volatility dynamics, trading and investing in the markets, or supervising and regulating the financial system. On the contrary, forecasts based on macro-shocks with a one- or three-month lag cannot reflect the up-to-date influence which economic fundamentals exert on financial markets. The use of the high-frequency macro domain in volatility modeling becomes even more crucial in crisis periods where the macro-conditions change very rapidly. Therefore, we explore the response of emerging equity markets to the unprecedented pandemic shock we experience nowadays apart from the global financial turmoil of the previous decade. The Covid-19 outbreak can be characterized as a generational phenomenon leading to a unique crisis. We are seeing a global socio-economic meltdown that quickly unfolds following the explosive Covid-related new deaths or infections and the unprecedented ways policymakers have responded. These are uncharted waters, and thus it is crucial that our macro-informed financial volatility modeling approach should rely on the highest possible frequency of economic news affecting the markets and not on data releases that refer to the previous month or quarter. The bivariate system of the two volatility equations is ready-to-use not only on stock market returns but also on further asset classes or financial instruments (e.g. exchange rate, cryptocurrency, commodity, real estate, and bond returns, associating them with alternative macro-proxies besides uncertainty) and multiple financial economics applications of business operations, such as bonds investing, foreign exchange trading and commodities hedging, core daily functions in the treasuries of most financial and non-financial corporations.

Overall, our volatility framework improves the HEAVY model and beats the standard variance specifications (GARCH, ARFIMA, and HAR models), with significant implications for market practitioners and policymakers on forecasting the financial returns’ second moment. Volatility modeling and forecasting are essential for asset valuation and risk management strategies. A reliable volatility forecast, exploiting in full the high-frequency domain and the macro-financial linkages, is the input variable of paramount importance for the processes of derivatives pricing, effective cross-hedging, Value-at-Risk measurement, investment allocation, portfolio optimization with different asset classes and financial instruments. Moreover, the robust volatility modeling approach we apply provides a useful tool not only for market players but also for policymakers. Policymaking includes continuous oversight duties and prudential regulation practices. Thus, it is imperative for the authorities to account for the volatility of financial markets across every aspect of the financial system’s policy responses, both post-crisis through stabilization policy reactions and pre-crisis through proactive assessment of financial risks. Regulators in emerging economies should consider, among the threats of their financial markets, the global destabilizing factors, beyond the local characteristics. In particular, the significant repercussions of uncertainty about US economic policies constitute the focus of attention in political debates, nowadays, with the widespread anxiety of world market players immediately after Trump’s victory and inauguration. The trade war uncertainty from the very early days of Trumponomics (e.g. steel and aluminum unconventional import tariffs) was followed by the overall fear of economic agents about future government initiatives. More intriguingly, our study is relevant to a highly topical issue nowadays, the Coronavirus pandemic, and contributes to the rapidly growing literature on the pandemic’s socio-economic effects and policy responses.

The remainder of the paper is structured as follows. Section 2 reviews the relevant literature. In Sect. 3, we detail the HEAVY formulation enriched with asymmetries, power transformations, and macro-effects. Section 4 describes the data and Sect. 5 presents the results for the benchmark, the macro-augmented asymmetric power models, and the crisis effect on volatility modeling parameters. In Sect. 6, we compute the multiple-step-ahead forecasts to measure the out-of-sample performance of the various specifications. The following Section focuses on the uncertainty effects across the parameters of the HEAVY specification and Sect. 8 discusses the policy implications of our findings. Finally, Sect. 9 concludes the analysis.

## Literature review

There is a large body of literature focusing on modeling and forecasting realized volatility, applying non-parametric estimation methods to high-frequency data. Following the first studies that formalized the daily realized measures on intra-daily returns (e.g. the realized variance established by Andersen et al. 2001, and the realized kernel by Barndorff-Nielsen et al. 2008), Andersen et al. (2001) and Corsi (2009) proposed long memory models for the conditional mean of the realized variance, that is the ARFIMA and HAR-RV specifications, respectively. In order to improve the forecasting accuracy of the various volatility models, econometricians have developed specifications combining daily with intra-daily measures. Engle (2002) estimated the daily GARCH-X specification adding the realized measure as an exogenous variable in the GARCH(1, 1) equation to capture the incremental information of the higher-frequency domain. Accordingly, Shephard and Sheppard (2010) introduced the HEAVY framework and Hansen et al. (2012) followed with the Realized GARCH (see also Barunik et al. 2016). Both HEAVY and Realized GARCH jointly estimate daily returns’ conditional variance and realized measure’s conditional mean. The HEAVY system of equations is proved to adopt to information arrival more rapidly than the classic daily GARCH process. A key advantage is the system’s robustness to certain forms of structural breaks, especially during crisis periods, since the mean reversion and short-run momentum effects result in higher quality performance in volatility level shifts and more reliable forecasts (Shephard and Sheppard 2010).

The financial econometrics literature on realized volatility dynamics mostly ignores important macro-factors that may affect the volatility pattern in the high-frequency domain. The empirical evidence on the economic drivers of equity volatility mostly employs lower- than daily-frequency macro-variables (monthly or quarterly). The first studies that explained monthly stock volatility with the business cycle dynamics were Schwert (1989) and Hamilton and Lin (1996). Engle and Rangel (2008) and Engle et al. (2013) use Spline- and MIDAS (Mixed-Data Sampling)—GARCH to link daily volatility with lower-frequency macro-proxies through the mixed-frequencies approach. Corradi et al. (2013) explore the economic impact on monthly returns, volatilities, and volatility risk-premia. Finally, Conrad and Loch (2015) test quarterly macro-regressors of daily conditional variance, and Meligkotsidou et al. (2019) include monthly macro-financial factors in monthly realized volatility quantile forecasting. The common finding of this area of research is the counter-cyclical behavior of financial volatility vis-a-vis economic activity indices. The economic intuition underpinning the link between equities and the macro-environment can be described as follows: equities volatility is tightly related to the uncertainty over the future expected cash flows of the firms issuing the stocks traded. These cash flows constitute the direct result of the firms’ performance which is, in turn, strongly affected by the business cycle dynamics. Besides the cash flow volume, the economic stance also determines the valuation of the cash flows through the discount rates used to define their present value (for a more detailed discussion on the economic theory supporting the stock volatility-macro conditions countercyclical nexus see Paye 2012; Christiansen et al. 2012, and Mittnik et al. 2015). Therefore, volatility modeling practice should rely on macroeconomic condition metrics to demonstrate the macro-financial effects on stock market volatility driven either by the cash flow or the discount rates channel.

Moreover, the unprecedented economic impact of the current pandemic and the high speed with which the crisis is evolving introduce uncertainty into models that assess the disastrous effects of the virus spread (Baker et al. 2020c). Caggiano et al. (2020) have predicted a huge decrease in the world output due to the Covid-induced uncertainty shock. Baker et al. (2020b) are the first to quantify this Covid-induced economic uncertainty combining three sources: stock market volatility, newspaper-based and business expectations survey-based uncertainties. Baker et al. (2020a) have investigated the disease’s detrimental impact on stock markets and demonstrate that the Covid-19 stock market effects have been by far more powerful than those of previous diseases (e.g. Spanish flu) due to the current pandemic’s severity, the more rapid diffusion of pandemic news, and the tighter macro-financial cross-border interconnectedness in the current globalization era. Turning to the pandemic shock on stock market volatility, Wang et al. (2020) have applied the HAR-RV (Heterogeneous Autoregressive-Realized Variance) model to predict daily stock market volatility during the Covid outbreak period. They extended the HAR equation with two alternative daily US uncertainty proxies: (i) the VIX index, which is the implied volatility metric of S&P500 used as a financial uncertainty source, and (ii) the US Economic Policy Uncertainty. Their forecasting exercise for nineteen equity indices indicates which uncertainty proxy produces more accurate forecasts for each market during the current crisis. Wang et al. (2020) investigated the US uncertainty spillovers on financial volatility across the different markets globally while the present paper focuses on the US uncertainty spillover effect alongside other US and global macro-factors on emerging stock market volatilities applying the sophisticated HEAVY framework for both returns and realized dispersion measures. Our macro-augmented specification with daily macro-proxies driving the volatility pattern during the last two decades with the 2008 turmoil and the pandemic period included, also advances the volatility modeling research which does not consider significant macro-determinants of the volatility process in the high-frequency domain.

In the present paper, we focus on the crucial role of economic uncertainty, besides other macro-variables, in volatility predictions applying the news-based Economic Policy Uncertainty index, the only macroeconomic uncertainty measure with a daily frequency provided by Baker et al. (2016) for the United States and the United Kingdom. The widely-recognized main advantage of the EPU index is its inclusivity since it incorporates both economic and policy-related factors giving rise to uncertainty. We investigate the effect of daily US EPU on emerging equity volatility modeling and its impact during the financial and health crises. Exploring the effects of uncertainty on financial volatility is very topical in the aftermath of the global financial crisis of 2007/08, since there has been renewed interest in this ‘amorphous’ concept (Bloom 2014). Based on the Knightian uncertainty definition (Knight 1921) and the seminal papers on uncertainty by Bernanke (1983) and Dixit and Pindyck (1994), researchers have focused on measuring this latent variable affecting the decision-making process by economic agents (Bekaert et al. 2013; Jurado et al. 2015; Mumtaz and Theodoridis 2018; Carriero et al. 2018; Jo and Sekkel 2019). Knight observed that households’ saving and consumption, corporations’ recruitments, investing and funding, traders’ portfolio selection, and regulators’ policy choices are deeply influenced by their ‘inability to forecast the likelihood of events happening’ (Bloom 2014). There is ample evidence that uncertainty disrupts the real economy (Colombo 2013; Jones and Olson 2013; Caggiano et al. 2017; Connolly et al. 2018; Tarassow 2019) through its effects on financial markets, namely by restraining economic agents from doing business due to their loss of confidence. At times of high uncertainty or lower confidence, individuals forsake consumption and turn to more savings while corporations restrict new investment projects or new recruitments (Gulen and Ion 2015). Further, asset market participants become more cautious, asset prices fall (either through the discount rate or the cash flow channel), volatilities and correlations soar (Pastor and Veronesi 2012, 2013; Li et al. 2015; Kelly et al. 2016; Bernal et al. 2016; Andreasson et al. 2016). A higher risk premium increases the cost of capital and generally the corporate funding costs (Alessandri and Mumtaz 2019) and erodes confidence in the financial system (see also Wisniewski and Lambe 2015; Bordo et al. 2016; Boumparis et al. 2017; Caliendo et al. 2018).

Despite the substantial advances in uncertainty research, the literature on the realized volatility dynamics of high-frequency financial variables associated with uncertainty is still in its infancy. Reviewing the few commendable attempts to explain the behavior of stock market volatility with EPU, we can trace back this link to Pastor and Veronesi (2013), who were the first to connect stock markets with monthly EPU using simple OLS regressions of monthly stock returns, volatilities, and correlations (unconditional) on the EPU index, whose parameter sign was consistently positive for correlations and volatilities and negative for returns. Antonakakis et al. (2013) further compute the dynamic conditional correlations between EPU, S&P 500 Stock index returns, and implied volatility (VIX) pairwise on a monthly frequency. The EPU–VIX correlation is positive and the EPU-returns negative, as expected, since elevated uncertainty depresses stock market performance and goes alongside higher stock market volatility. More recently, Fang et al. (2018) have related daily gold futures volatility with the monthly Global EPU index through the GARCH-MIDAS framework. They provide evidence to support the strong positive effect of uncertainty on gold volatility and its power in forecasting the monthly realized volatility of gold futures. Finally, Cho et al. (2018) highlight the fact that high exchange rate volatility is linked with elevated EPU leading to carry trade losses.

## The econometric framework

Building on the benchmark HEAVY model of Shephard and Sheppard (2010) who combine two volatility estimators in a bivariate system, we apply the HEAVY extension which accounts for downside risk (asymmetries), power terms, and economic effects, and estimate an augmented version including these three additional features to improve volatility modeling and forecasting.

### The benchmark specification

The HEAVY model uses two variables: the close-to-close stock returns ($r_{t}$) and the realized measure of variation based on high-frequency data, ${\textit{RM}}_{t}$. We first calculate the signed square rooted (SSR) realized measure as follows: $\widetilde{{\textit{RM}}_{t}}=\textit{sign}(r_{t})\sqrt{{\textit{RM}}_{t}}$, where *sign*$(r_{t})=1$, if $r_{t}\geqslant 0$ and *sign*$(r_{t})=-1$, if $r_{t}<0$.

We assume that the returns and the SSR realized measure are characterized by the following relations:

$$\begin{aligned} r_{t}=e_{rt}\sigma _{rt},\quad \widetilde{{\textit{RM}}_{t}}=e_{Rt}\sigma _{Rt}, \end{aligned}$$

where the stochastic term $e_{it}$ is independent and identically distributed (*i.i.d*), $i=r,R$; $\sigma _{it}$ is positive with probability one for all *t* and it is a measurable function of ${\mathcal {F}} _{t-1}^{(XF)}$, that is the filtration generated by all available information through time $t-1$. We will use ${\mathcal {F}}_{t-1}^{(HF)}$ ($X=H$) for the high-frequency past data, i.e., for the case of the realized measure, or ${\mathcal {F}}_{t-1}^{(LoF)}$ ($X=Lo$) for the low-frequency past data, i.e., for the case of the close-to-close returns. Hereafter, for notational convenience, we will drop the superscript *XF*.

In the HEAVY/GARCH model $e_{it}$ has zero mean and unit variance. Therefore, the two series have zero conditional means and their conditional variances are given by

$$\begin{aligned} {\mathbb {E}}(r_{t}^{2}\left| {\mathcal {F}}_{t-1}\right. )=\sigma _{rt}^{2} \quad \text {and}\quad {\mathbb {E}}(\widetilde{{\textit{RM}}_{t}}^{2}\left| {\mathcal {F}} _{t-1}\right. )={\mathbb {E}}({\textit{RM}}_{t}\left| {\mathcal {F}}_{t-1}\right. )=\sigma _{Rt}^{2}, \end{aligned}$$

where ${\mathbb {E}}(\cdot )$ denotes the expectation operator. The returns equation is called HEAVY-*r* and, similarly, the realized measure equation is denoted as HEAVY-*R*.

### The macro-augmented asymmetric power model

The asymmetric power (AP) specification for the HEAVY(1, 1) model consists of the following equations (in what follows, for notational simplicity, we drop the order of the model if it is (1, 1)):

1 $$\begin{aligned} (1-\beta _{i}L)(\sigma _{it}^{2})^{\frac{\delta _{i}}{2}}=\omega _{i}+(\alpha _{ir}+\gamma _{ir}s_{t-1})L(r_{t}^{2})^{\frac{\delta _{r}}{2} }+(\alpha _{iR}+\gamma _{iR}s_{t-1})L({\textit{RM}}_{t})^{\frac{\delta _{R}}{2}}, \end{aligned}$$

where *L* is the lag operator, $\delta _{i}$ $\in {\mathbb {R}}_{>0}$ (the set of the positive real numbers), for $i=r,R$, are the power parameters and $s_{t}=0.5[1-\textit{sign}(r_{t})]$, that is, $s_{t}=1$ if $r_{t}<0$ and 0 otherwise; $\gamma _{ii}$, $\gamma _{ij}$ ($i\ne j$) are the own and cross leverage parameters, respectively^2^; positive $\gamma _{ii}$, $\gamma _{ij}$ means a larger contribution of negative ‘shocks’ in the volatility process. In this specification the powered conditional variance, $(\sigma _{it}^{2})^{\delta _{i}/2}$, is a linear function of the lagged values of the powered transformed squared returns and realized measure.

We will distinguish between three different asymmetric cases: the double one (DA: $\gamma _{ij}\ne 0$ for all *i* and *j*) and two more, own asymmetry (OA: $\gamma _{ij}=0$ for $i\ne j$ only) and cross asymmetry (CA: $\gamma _{ii}=0$).

The $\alpha _{iR}$ and $\gamma _{iR}$ are called the (four) Heavy parameters (own when $i=R$ and cross when $i\ne R$). These parameters capture the impact of the realized measure on the two conditional variances. Similarly, the $\alpha _{ir}$ and $\gamma _{ir}$ (four in total) are called the Arch parameters (own when $i=r$ and cross for $i\ne r$). They capture the influence of the squared returns on the two conditional variances.

The asymmetric power model is equivalent to a bivariate AP-GARCH process for the returns and the SSR realized measure (see, for example, Conrad and Karanasos 2010). If all four Arch parameters are zero, then we have the AP version of the benchmark HEAVY specification, where the only unconditional regressor is the first lag of the powered ${\textit{RM}}_{t}$.

Furthermore, we should mention that all the parameters in this bivariate system should take non-negative values (see, for example, Conrad and Karanasos 2010). We extend the realized measure equation with the non-negative macro-proxies: the volatility of US Economic Policy Uncertainty, ${\textit{EPUvol}}_{t}$, the US financial uncertainty (the S&P 500 implied volatility index), ${\textit{VIX}}_{t}$, the Bond market (the Merrill Lynch MOVE treasury bonds implied volatility index or the Moody’s BAA over AAA corporate bonds spreads), ${\textit{BO}}_{t}$, the Commodity benchmarks (the S&P GSCI all commodities index or the S&P GSCI crude oil index prices), ${\textit{CO}}_{t}$, and the infectious disease news effect on stock markets, ${\textit{ID}}_{t}$. The macro-augmented (m) AP-HEAVY system is characterized by the following equation for the realized measure:

2 $$\begin{aligned} (1-\beta _{R}L)(\sigma _{Rt}^{2})^{\frac{\delta _{R}}{2}}&=\omega _{R}+(\alpha _{Rr}+\gamma _{Rr}s_{t-1})L(r_{t}^{2})^{\frac{\delta _{r}}{2} }+(\alpha _{RR}+\gamma _{RR}s_{t-1})L({\textit{RM}}_{t})^{\frac{\delta _{R}}{2}} \nonumber \\&\quad +\phi _{R}{\textit{EPUvol}}_{t-1}+\lambda _{R}{\textit{VIX}}_{t-1}+\zeta _{R}{\textit{BO}}_{t-1}+\vartheta _{R}{\textit{CO}}_{t-1}+\eta _{R}{\textit{ID}}_{t-1}. \end{aligned}$$

Equation (2) incorporates four Macro-parameters, $\phi _{R}$, $\lambda _{R}$, $\zeta _{R}$, $\vartheta _{R}$, and $\eta _{R}$, which capture the macro-effects on the power transformed realized measure. The returns equation remains the same as in the non-augmented specification, without the direct effect from the macro-variables ($\phi _{r},\lambda _{r},\zeta _{r},\vartheta _{r},\eta _{r}=0$).

To sum up, the benchmark model is characterized by two conditional variance equations, the GARCH(1, 0)-X formulation for returns and the GARCH(1, 1) formulation for the SSR realized measure:

$$\begin{aligned} \text {HEAVY-}r&\text {:} (1-\beta _{r}L)\sigma _{rt}^{2}=\omega _{r}+\alpha _{rR}L({\textit{RM}}_{t}), \\ \text {HEAVY-}R&\text {:} (1-\beta _{R}L)\sigma _{Rt}^{2}=\omega _{R}+\alpha _{RR}L({\textit{RM}}_{t}) \end{aligned}$$

Equation (2) gives the general formulation of our macro-augmented extension for ${\textit{RM}}_{t}$, which adds asymmetries and power transformations to the benchmark specification (see also the Supplementary Appendix for our theoretical considerations). We use the existing Gaussian quasi-maximum likelihood estimators (QMLE) and multistep-ahead predictors already applied (Ding et al. 1993) in the APARCH framework (see, for example, He and Teräsvirta 1999; Laurent 2004; Karanasos and Kim 2006). We will first estimate both conditional variance equations in the general form with all Heavy, Arch, and their Asymmetry parameters given by Eq. (2) and in case a parameter is insignificant, we will exclude it and this will result in a reduced form that is statistically preferred for each volatility process. For example, in the returns and realized measure conditional variances estimation, the own and cross Arch parameters ($\alpha _{rr}$ and $\alpha _{Rr}$ respectively) prove to be insignificant and, therefore, are excluded (see Sect. 5.2, Table 3, Panels A and B) since this is the way to reach the returns and realized measure formulations that are statistically preferred.

## Data description

The HEAVY framework is estimated for five emerging stock index returns and realized volatilities. According to the analysis in Shephard and Sheppard (2010), this formulation considerably improves the volatility modeling by allowing momentum and mean reversion effects and adjusting quickly to the structural breaks in volatility. We extend their benchmark specification, by adding the features of power transformed conditional variances, leverage, and macro-effects in the volatility process.

### Stock index data

We use daily data for two American and three Asian stock market indices extracted from the Oxford-Man Institute’s (OMI) realized library version 0.3 (Heber et al. 2009): Brazil’s Bovespa index (BRAZIL) and Mexico’s IPC index (MEXICO) from the Americas, Shanghai Composite index (CHINA), India’s Nifty 50 index (INDIA), and South Korea’s KOSPI index (KOREA) from Asia. We choose the particular emerging markets due to data (stock index and sample size) availability in the OMI realized library. Our sample covers the period from 03/01/2000 to 30/11/2020 and three out of five indices belong to the BRICS group of emerging markets (Brazil, India, and China). Regarding our research interest in the two Americas’ emerging markets, Brazil plays a dominant economic role internationally and Mexico is the second largest economy in Latin America. For the three Asian countries included, China and Korea are considered among the fastest growing economies while Indian stock markets have progressively undergone a fundamental financial liberalization process since early 1990s.

The OMI’s realized library includes daily stock market returns and several realized volatility measures calculated on high-frequency data from the Reuters DataScope Tick History database. The data are first cleaned and then used in the realized measures calculations (see also the library’s documentation in Heber et al. 2009). We use the daily closing prices, $$ P_{t}^{C}$$, to form the daily returns as follows: $$r_{t}=[\ln (P_{t}^{C})-\ln (P_{t-1}^{C})]\times 100$$, and two realized measures as drawn from the library: the 5-min realized variance and the realized kernel. The estimation results using the two alternative measures are very similar, so we present only the ones with the realized variance (the results for the realized kernel are available upon request). Table 1 presents the five stock indices extracted from the database and provides volatility estimations for each one’s squared returns and realized variances time series for the respective sample period (see also the stock index series graphs in the Supplementary Appendix, Figure E.1). We calculate the standard deviation of the series and the annualized volatility. Annualized volatility is the square rooted mean of 252 times the squared return or the realized variance. The standard deviations are always lower than the annualized volatilities. The realized variances have lower annualized volatilities and standard deviations than the squared returns since they ignore the overnight effects and are affected by less noise. The returns represent the close-to-close yield and the realized variances the open-to-close variation. The annualized volatility of the realized measure is between 15 and 20%, while the squared returns show figures from 20 to 29%.

**Table 1:** Data description

Index	Total sample period			$$ r_{t}^{2}$$		$$RV_{t}$$	
	Start date	End date	Obs.	Avol	SD	Avol	SD
BRAZIL	03/01/2000	30/11/2020	5146	0.290	0.098	0.199	0.028
MEXICO	03/01/2000	30/11/2020	5243	0.203	0.045	0.145	0.017
CHINA	04/01/2000	30/11/2020	5049	0.246	0.063	0.201	0.027
INDIA	03/01/2000	27/11/2020	5185	0.233	0.074	0.181	0.042
KOREA	04/01/2000	30/11/2020	5146	0.236	0.068	0.174	0.022

The table reports the sample period (start and end date) and the number of observations (Obs.) of each stock index covered in this study. The reported statistics include the annualized volatility (Avol) and the standard deviation (SD) of the stock index squared returns ($r_{t}^{2}$) and realized variance (${\textit{RV}}_{t}$). BRAZIL stands for Brazil’s Bovespa index, MEXICO for Mexico’s IPC index, CHINA for the Shanghai Composite index, INDIA for India’s Nifty 50 index, and KOREA for South Korea’s KOSPI index

### Macroeconomic proxies

In order to shed light on the macro-financial linkages, we augment the financial volatility HEAVY process with five non-negative macro-proxies of daily frequency. Research on the economic drivers of financial volatility lacks evidence on daily macro-factors of the daily or intra-daily stock index volatility pattern. Motivated by this literature gap, we augment the model of both daily and intra-daily volatility with daily macro-variables that proxy the business cycle conditions used in the existing monthly or quarterly studies of volatility determinants. In line with Conrad and Loch (2015), we proxy the macroeconomic environment through economic activity, monetary and business conditions, and sentiment daily variables that could explain stock index realized variance. Since GDP, industrial production, unemployment, inflation, consumer sentiment, or any commonly-used activity, monetary base, and sentiment index are not measured with a daily frequency, we turn to relevant daily variables. The EPU index is directly related to the business cycle with significant contractive effects on investment and employment (Baker et al. 2016). It is used here in place of the activity variables included in all prior studies. We expect the opposite sign effect from the sign previously observed for economic activity variables since uncertainty is negatively correlated to activity and higher uncertainty is strongly associated with recessions. The EPU index applied is also considered as an alternative to sentiment and macroeconomic volatility (Conrad and Loch 2015). The S&P 500 implied volatility index is our financial uncertainty proxy (VIX index in Corradi et al. 2013). Daily credit condition variables are chosen to account for the business and monetary conditions’ impact on financial volatility, following Schwert (1989), who uses financial leverage variables, interest rate and corporate bond returns volatility. We further use daily commodity price indices motivated by the fact that commodity price increases and oil, in particular, are often associated with recessions in the macroeconomy (Barsky and Kilian 2004). Therefore, we expect a significant surge in stock market volatility following a rise in commodity prices, which has proved harmful for real economic activity. Lastly, we consider the infectious disease news effect on equity market volatility predicting a positive relationship with emerging markets variability.

Our first macro-variable, the news-based Economic Policy Uncertainty index is established by Baker et al. (2016) and retrieved from http://www.policyuncertainty.com/. The site, maintained by Baker, Bloom, and Davis, provides daily EPU data for the US starting from 1985. The EPU index effectively captures the broad ‘amorphous’ concept of economic uncertainty (Bloom 2014). The 2008 global financial crisis has brought the previously overlooked notion of economic uncertainty to the frontline of academics’, policymakers’ and practitioners’ interest. We are now witnessing an extensive burgeoning literature with uncertainty as its principal topic and exploring the widely-recognized countercyclical uncertainty effects on macroeconomic and financial indicators across the business cycle. In particular, for unique crisis events and long-lasting recession periods, academics try to scrutinize all possible factors from their arsenal of indicators, which could prove to be forces behind the poor economic performance. Uncertainty in the agents’ thoughts has been recently verified as a crucial factor explaining a substantial part of economic fluctuations. Our motivation and recognition of the relative merits of the news-based EPU metric are discussed in the literature review (Sect. 2). We further include one more US factor for financial uncertainty, the VIX index of S&P 500 implied volatility.

Moving to the credit market conditions, we use two alternative Bond market global benchmarks: the Merrill Lynch MOVE 1 month index (MOVE) and the Moody’s corporate bonds default spread, defined as the difference of BAA over AAA bond yields (BAA_AAA). The MOVE index is an estimate of the option implied volatility of US treasury bonds. It is the treasury counterpart of the ‘fear’ index (VIX) for S&P 500 and captures the sovereign credit market stance. Higher sovereign bond volatility denotes higher turbulence in the credit channel for sovereigns with direct spillovers to financial and non-financial corporations’ credit conditions. The Moody’s index provides daily averages of global AAA and BAA corporate bond yields (higher spreads denote higher credit risk pricing for corporations, that is higher cost of financing). The Moody’s default spread is used as an alternative to the MOVE index for the credit channel. Moreover, the Commodity market conditions are proxied by two alternative global factors: the S&P GSCI index (GSCI) and the S&P GSCI Crude Oil subindex (GSCI_OIL). Both capture the cost of production for firms in the economy, where rising commodity values can lead to production and investment deterioration due to increased cost effects on economic activity. On the one hand, the S&P Goldman Sachs Commodity Index (all commodities included) is the widely-recognized commodity markets performance benchmark. On the other hand, GSCI crude oil subindex represents the most important commodity as an energy source across all economies. The GSCI_OIL is used as our alternative macro-regressor to the GSCI, where, besides oil, most liquid commodities are incorporated. VIX and the four bonds and commodities variables are retrieved from Thomson Reuters Datastream. Finally, we include the infectious disease detrimental impact on equity market volatility proxied by the Infectious Disease Equity Market Volatility Tracker (ID_EMV) of Baker et al. (2020a). ID_EMV is a newspaper-based metric (available at https://www.policyuncertainty.com) that quantifies the crucial role of news about infectious diseases (e.g. epidemics/pandemics, MERS, SARS, H1N1, Covid-19, etc.) on US stock market volatility. Given that our sample covers the Coronavirus pandemic crash, we expect a significant impact on realized volatility from disease outbreaks at least during the current health crisis.

**Fig. 1:**
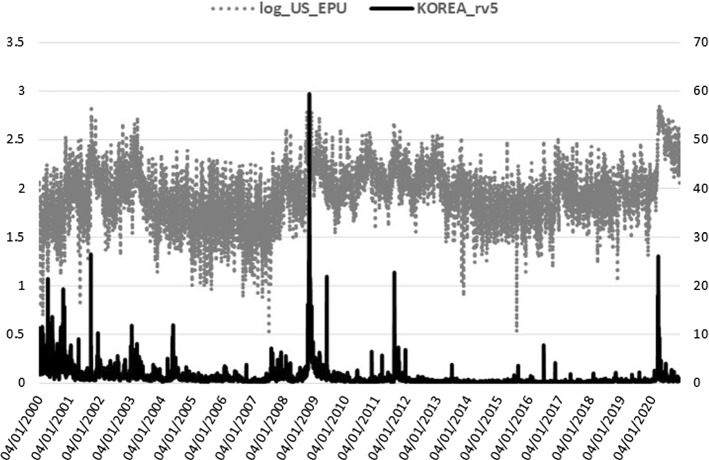
US EPU and KOSPI Realized Variance

**Fig. 2:**
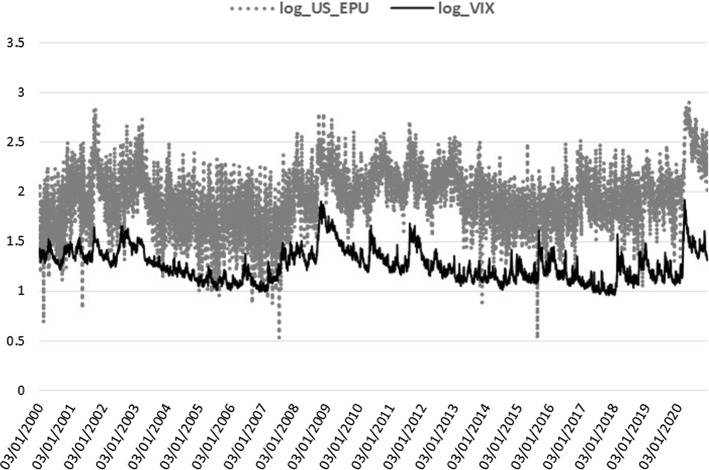
US EPU and Financial uncertainty

**Fig. 3:**
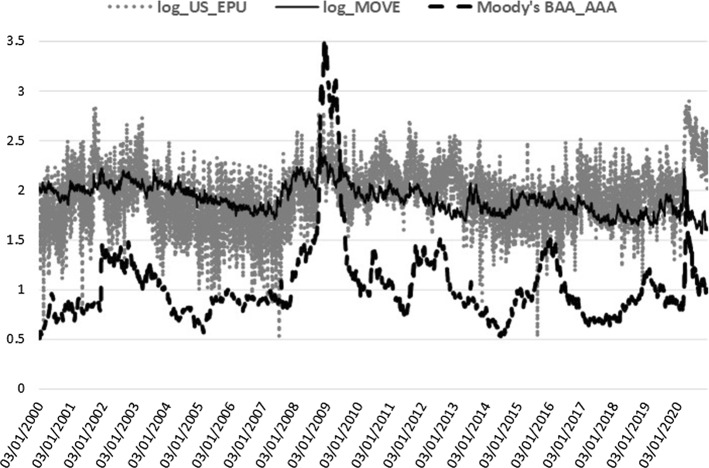
US EPU and the Credit market proxies

**Fig. 4:**
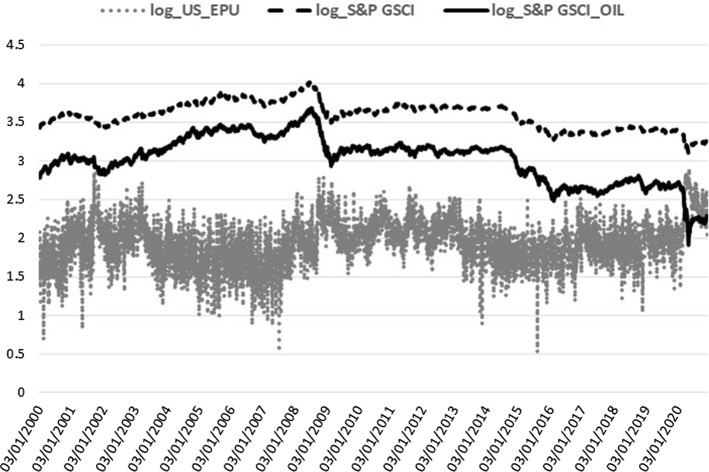
US EPU and the Commodity market proxies

**Fig. 5:**
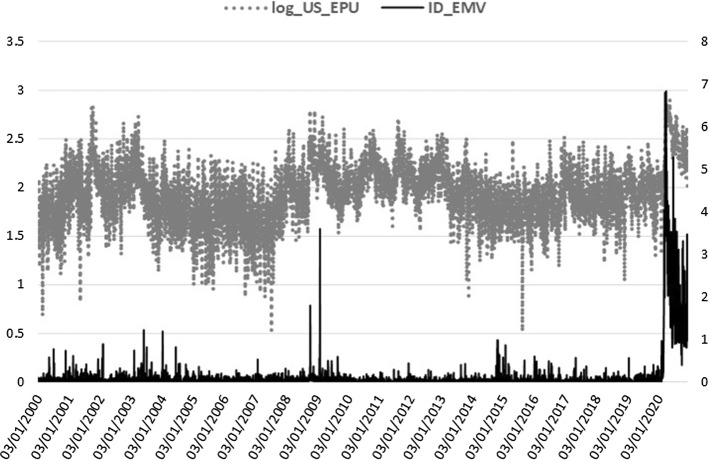
US EPU and the Infectious disease news impact on equity volatility

VIX, MOVE, GSCI, and GSCI_OIL are log-transformed and together with the BAA_AAA spread and ID_EMV level, they are all included in the realized measure equation, where they are shown to be jointly significant. The squared return series of the EPU index, signifying the volatility of EPU (EPUvol), is added in the realized measure equation to estimate the direct EPU volatility spillover effect, whereas for the indirect effect we use the log-level of EPU^3^ (see Sect. 7). In the macro-augmentation of the HEAVY model, we are restricted to using only non-negative variables with estimated parameters of positive sign due to the GARCH positivity constraints. Consequently, we focused our analysis of the macro-financial linkages on the EPU and VIX indices for uncertainty, the four bonds and commodities variables, and the disease proxy, which are characterized by non-negative values only and exert an inflating impact on realized volatility. Increased uncertainty, bond spreads and volatility, commodity prices, and disease impact, all contribute to financial volatility heightening, apparent especially during economic downturns. Figures 1, 2, 3, 4 and 5 clearly show that higher realized volatility is observed mainly in times of elevated uncertainty, credit market turbulence, boosts in commodity prices, and disease outbreaks (mostly during the Covid-19 period).

Before selecting the five macro-financial regressors for the realized variance equation, we tested a plethora of real activity, monetary, and financial candidates in daily frequency as discussed in the relevant literature. We chose the combination of jointly significant variables that minimize the information criteria and maximize the log-Likelihood score. Given the GARCH positivity constraints, we had to exclude the variables with negative values and the variables with a negative impact on volatility. For example, confidence indices (e.g. the daily News Sentiment Index-NSI from the FRB San Francisco dataset, Buckman et al. 2020) were excluded given their negative-signed effect on volatility and replaced by the uncertainty proxies (the sentiment antipode of confidence). Furthermore, the 3-month libor and treasury bill yields, as well as the daily global Financial Stress Index-FSI of the Office of Financial Research (OFR) could not be included as credit condition proxies since their time series take negative values. We additionally tested a non-negative proxy of the real estate market (the log-transformed Dow Jones [DJ] REIT index). This proved to be highly significant but we should exclude it since the negative sign of the relevant parameter violates our econometric framework constraints. Better performance of the real estate sector is associated with higher REIT’s level mostly in economic growth periods and is consistently negatively related to financial volatility. Finally, the realized variance receives sound negative impact from two economic activity indicators with values not bounded to the positive territory of real numbers and, therefore, they have been excluded. We used the Aruoba–Diebold–Scotti (ADS) Business Conditions index (Aruoba et al. 2009) and the Yield Curve slope, which are among the unique economic activity indicators available on a daily frequency. The ADS index tracks daily real business conditions based on economic data releases and the Yield Curve slope, as calculated by the difference of the 10-year minus the 3-month treasury bond yields, and it has proved to be a powerful predictor of future economic activity (Estrella and Hardouvelis 1991). Financial volatility receives a significant negative effect from both variables, as expected since lower ADS and term structure slope values indicate economic worsening associated with higher stock market volatility. This opens several paths for future research on macro-financial linkages in the high-frequency domain to connect real activity variables (such as DJ REIT, ADS, Yield Curve slope), excluded here, with realized variation measures in the absence of positivity constraints of the econometric framework applied.^4^

## Empirical findings

Building upon the introduction of the GARCH-X process by Engle (2002) to include realized measures as exogenous regressors in the conditional variance equation, Han and Kristensen (2014) and Han (2015) studied the asymptotic properties of this new specification with a fractionally integrated (nonstationary) process included as covariate (see also Francq and Thieu 2019). Moreover, Nakatani and Teräsvirta (2009) and Pedersen (2017) focused on the multivariate case, the so-called extended constant conditional correlation, which allows for volatility spillovers and they developed inference and testing for the QMLE parameters (see also Ling and McAleer 2003 for the asymptotic theory of vector ARMA-GARCH processes). For the extended HEAVY models, we employ the existing Gaussian QMLE and multistep-ahead predictors applied in the APARCH framework (see, for example, He and Teräsvirta 1999; Laurent 2004; Karanasos and Kim 2006). Following Pedersen and Rahbek (2019), we first test for arch effects and after rejecting the conditional homoscedasticity hypothesis we apply one-sided significance tests of the covariates added to the estimated GARCH processes.

### The benchmark HEAVY results

Within the HEAVY framework, we first estimate the benchmark formulation as in Shephard and Sheppard (2010), that is, without asymmetries, power transformations, and macro-effects, obtaining very similar results (Table 2). The only unconditional regressor in both equations is the first lag of the ${\textit{RM}}_{t}$. In other words, the chosen returns equation is a GARCH(1, 0)-X process dropping out the own Arch effect, $\alpha _{rr}$, from lagged squared returns since it becomes insignificant when we add the cross effect of the lagged realized measure as regressor, with a Heavy parameter, $\alpha _{rR}$, high in value and significance across all indices. The momentum parameter, $\beta _{r}$, is estimated around 0.47–0.80. For the SSR realized variance, the best-chosen model is the GARCH(1, 1) without the cross effect from lagged squared returns. The Heavy term, $\alpha _{RR}$, is estimated between 0.32 and 0.50 and the momentum, $\beta _{R}$, is around 0.49–0.67. The benchmark HEAVY system of equations chosen, after testing all three alternative GARCH models of order (1, 1), (1, 1)-X, and (1, 0)-X, is the same as in Shephard and Sheppard (2010) with similar parameter values and the identical conclusion that the realized measure of variation does all the work at moving around the conditional variances of stock returns and the SSR realized variance. The benchmark’s conclusion, as we show in this study, does not hold for the more richly parametrized macro-augmented asymmetric power model. More importantly, according to the Sign Bias test (SBT) of Engle and Ng (1993), the asymmetric effect is obviously omitted from the benchmark specification with the sign parameter always significant (SBT p-values lower than 0.04).

**Table 2:** The benchmark HEAVY model

	BRAZIL	MEXICO	CHINA	INDIA	KOREA
*Panel A. Stock returns: HEAVY-r*					
$$(1-\beta _{r}L)\sigma _{rt}^{2}=\omega _{r}+\alpha _{rR}L({\textit{RM}}_{t})$$					
$$\beta _{r}$$	$$\underset{(0.080)^{***}}{0.63}$$	$$\underset{ (0.032)^{***}}{0.82}$$	$$\underset{(0.050)^{***}}{ 0.80}$$	$$\underset{(0.070)^{***}}{0.47}$$	$$\underset{(0.059)^{***}}{0.64}$$
$$\alpha _{rR}$$	$$\underset{(0.131)^{***}}{0.65}$$	$$\underset{ (0.053)^{***}}{0.28}$$	$$\underset{(0.088)^{***}}{ 0.28}$$	$$\underset{(0.098)^{***}}{0.64}$$	$$\underset{(0.109)^{***}}{0.67}$$
SBT	$\underset{[0.00]}{2.69}$	$\underset{[0.00]}{4.99}$	$\underset{ [0.00]}{2.99}$	$\underset{[0.02]}{2.27}$	$\underset{ [0.04]}{2.03}$
$${\textit{lnL}}$$	$${-9781.47}$$	$${-8051.11}$$	$${-8573.58}$$	$$ {-8517.40}$$	$${-8054.15}$$
*Panel B. Realized measure: HEAVY-R*					
$$(1-\beta _{R}L)\sigma _{Rt}^{2}=\omega _{R}+\alpha _{RR}L({\textit{RM}}_{t})$$					
$$\beta _{R}$$	$$\underset{(0.051)^{***}}{0.55}$$	$$\underset{ (0.055)^{***}}{0.67}$$	$$\underset{(0.041)^{***}}{ 0.49}$$	$$\underset{(0.046)^{***}}{0.52}$$	$$\underset{(0.037)^{***}}{0.53}$$
$$\alpha _{RR}$$	$$\underset{(0.045)^{***}}{0.41}$$	$$\underset{ (0.059)^{***}}{0.32}$$	$$\underset{(0.042)^{***}}{ 0.50}$$	$$\underset{(0.048)^{***}}{0.47}$$	$$ \underset{(0.041)^{***}}{0.47}$$
SBT	$\underset{[0.00]}{3.08}$	$\underset{[0.00]}{4.90}$	$\underset{ [0.01]}{2.50}$	$\underset{[0.02]}{2.37}$	$\underset{ [0.00]}{2.80}$
$${\textit{lnL}}$$	$${-7735.39}$$	$${-6183.46}$$	$${-7228.98}$$	$$ {-6733.72}$$	$${-6521.24}$$

The table reports the estimation results of the Benchmark HEAVY model for each stock index. HEAVY-*r* is the returns equation and HEAVY-*R* is the realized measure equation. SBT denotes the Sign Bias test of Engle and Ng (1993). $${\textit{lnL}}$$ denotes the log-Likelihood value for each specification. The numbers in parentheses are robust standard errors. $$^{***}$$, $$^{**}$$, $$^{*}$$ denote significance at the 0.01,  0.05,  0.10 level, respectively. The numbers in square brackets are p-values. BRAZIL stands for Brazil’s Bovespa index, MEXICO for Mexico’s IPC index, CHINA for the Shanghai Composite index, INDIA for India’s Nifty 50 index, and KOREA for South Korea’s KOSPI index

### The macro-augmented asymmetric power HEAVY results

Moving to the extension of the benchmark HEAVY system, Table 3 presents the estimation results for the chosen macro-augmented asymmetric power specifications. Wald and *t*-tests are used to test the significance of the Heavy and Arch parameters, rejecting the null hypothesis at 10% in all cases. We should highlight the fact that since all the parameters take non-negative values, we use one-sided tests (see, for example, Pedersen and Rahbek 2019). For both returns and realized variance, we statistically prefer either the double asymmetric power (DAP) specification since both power transformed conditional variances are significantly affected by own and cross asymmetries or the cross (own) asymmetric power—CAP (OAP)—model when only the cross (own) asymmetries are included. We estimate the power terms separately with a two-stage procedure, as follows: We, first, estimate univariate asymmetric power specifications for the returns and the realized measure. The Wald tests for the estimated power terms (available upon request) reject the hypotheses of $\delta _{i}=1$ and $\delta _{i}=2$ in most cases. In the second stage, we use the estimated powers, $\delta _{r}$ and $\delta _{R}$, from the first step to power transform each series’ conditional variance and incorporate them into the bivariate model. The sequential procedure produces the fixed power term values, which are the same for both specifications ($\delta _{r}$ and $\delta _{R}$ are common for Panels A and B).

For the returns (Panel A), the estimated power, $\delta _{r}$, lies between 1.30 and 1.60 (see Panel C). The Heavy cross effect parameter, $\alpha _{rR}$, is significant in most cases, except for Mexico. The Heavy cross asymmetry, $\gamma _{rR}$, is insignificant and excluded in China and India equations. Consequently, DAP returns specification is preferred for three out of five indices (Brazil, Mexico, Korea) and OAP is chosen for the other two cases (China, India). Although $\alpha _{rr}$ is insignificant and is excluded in all cases, the own asymmetry parameter is always significant with $\gamma _{rr}\in [0.05,0.14]$. In other words, the lagged values of both powered variables, that is, the realized measure and the squared negative returns, drive the model of the power transformed conditional variance of the returns. Moreover, the momentum parameter, $\beta _{r}$, is estimated to be around 0.77 to 0.93. All five indices generated very similar DAP/OAP specifications without macro-effects since we statistically prefer to include the macro-regressors in the realized measure equation. Similarly, for the realized measure the most preferred specification is the m-CAP one (for Mexico only, we choose the m-DAP model). The power, $\delta _{R}$, is estimated from 1.00 to 1.20 and is consistently lower than the returns power term (see Panel C). The Heavy parameter, $\alpha _{RR}$, is always significant and around 0.19 (min. value) to 0.40 (max. value), while the own asymmetry, $\gamma _{RR}$, appears only in the realized measure equation of Mexico (see Panel B). Moreover, the cross asymmetry Arch parameter is always significant with $\gamma _{Rr}\in [0.02,0.05]$. This means that the power transformed conditional variance of ${\widetilde{{\textit{RM}}}} _{t}$ is significantly affected by the lagged values of both powered variables: squared negative returns and realized measure. Further, the momentum parameter, $\beta _{R}$, is estimated to be around 0.52 to 0.73.

Lastly, the lagged macro-effects are highly significant, with the expected positive sign in all cases (see Panel B). The power transformed realized variance receives the boosting impact from higher volatility of the US EPU index in all but one case (China), $\phi _{R}\in [0.01,0.02]$, in line with Pastor and Veronesi (2013), who were the first to associate stock market volatilities with EPU, resulting in a positive link. The US financial uncertainty effect of VIX, $\lambda _{R}$, is significant only for Brazil and Korea. The uncertainty effects confirm the finding of Conrad and Loch (2015), among others, on the negative effect of consumer confidence (University of Michigan Consumer Sentiment index), which is the opposite sentiment to uncertainty and is estimated here with the expected opposite sign, as well. Regarding the bond and commodity markets, we prefer to use common global proxies across all emerging stock markets. Bond market conditions are captured by either the MOVE index (Brazil, Korea) or the Moody’s default spread (Mexico, China, India). Increased US treasury implied volatility or elevated corporate bond default spreads raise realized volatility in stock markets ($\zeta _{R}\in [0.01,0.06]$), as expected since the turbulence in the credit markets always gives significant volatility spillover effects to stock markets. Hereby, we confirm, among others, Engle and Rangel (2008), who estimate a positive effect of short-term government bond interest rate volatility on stock market volatility through the Spline-GARCH specification. Turning to commodities ($\vartheta _{R}\in [0.01,0.04]$), we prefer the GSCI all commodities index in three cases, while, for Mexico and India, the GSCI oil subindex is the chosen commodity regressor. Lower commodity prices mean decreased cost of supplies for firms in the economy, propelling productivity, investment, and, more generally, economic growth and, at the same time, reducing stock market volatilities. Given that increased oil prices are mostly coincident with recession periods (Barsky and Kilian 2004), the positive link of realized variance and commodity prices, captured by $\vartheta _{R}$, supports the negative association of economic activity with stock market volatility, in accordance with the existing literature. All prior volatility determinant studies have provided sound evidence on the negative sign effect of economic activity proxies on stock market volatility (see, for example, the GDP growth parameters in Engle and Rangel 2008). Finally, the coefficient of the fifth macro-regressor, $\eta _{R}$, is significant for three out of five cases with the ID_EMV index tracking the infectious disease news impact on US equity volatility and spreading the disease effect to Brazil’s, India’s, and Korea’s stock markets.

Overall, our results show strong Heavy effects (captured by the $\alpha _{rR}$, $\gamma _{rR}$, and $\alpha _{RR}$ parameters), as well as asymmetric Arch influences (the estimated $\gamma _{rr}$ and $\gamma _{Rr}$ are always significant) and macro-impacts (measured by $\phi _{R}$, $\lambda _{R}$, $\zeta _{R}$, $\vartheta _{R}$, and $\eta _{R}$). According to the log-Likelihood (${\textit{lnL}}$) values reported, the log-Likelihood is always higher for the m-DAP specifications compared to the benchmark one, that is without asymmetries, powers, and macro-effects, proving the superiority of our model’s in-sample estimation (see also the comparison of the two models in terms of the Bovespa standardized residuals graphs in the Supplementary Appendix, Figure E.2). The SBT statistics further show that the asymmetric effect is not omitted any more since the sign parameters are insignificant with p-values consistently higher than 0.17. Table 9 (in the Appendix) provides additional results for the realized measure equation with the DAP extension before including the macro-effects. We followed the particular stepwise estimation procedure before selecting our final chosen model with powers, asymmetries, and all five macro-factors.

**Table 3:** The m-DAP-HEAVY model

	BRAZIL	MEXICO	CHINA	INDIA	KOREA
*Panel A. Stock returns: m-DAP-HEAVY-r*					
$$(1-\beta _{r}L)(\sigma _{rt}^{2})^{\frac{\delta _{r}}{2} }=\omega _{r}+\gamma _{rr}s_{t-1}L(r_{t}^{2})^{\frac{\delta _{r}}{2} }+(\alpha _{rR}+\gamma _{rR}s_{t-1})L({\textit{RM}}_{t})^{\frac{\delta _{R}}{2}}$$					
$$\beta _{r}$$	$$\underset{(0.039)^{***}}{0.84}$$	$$\underset{ (0.091)^{***}}{0.93}$$	$$\underset{(0.059)^{***}}{ 0.79}$$	$$\underset{(0.019)^{***}}{0.87}$$	$$\underset{ (0.033)^{***}}{0.77}$$
$$\alpha _{rR}$$	$$\underset{(0.046)^{**}}{0.09}$$		$$\underset{ (0.060)^{***}}{0.20}$$	$$\underset{(0.016)^{***}}{ 0.08}$$	$$\underset{(0.041)^{***}}{0.21}$$
$$\gamma _{rr}$$	$$\underset{(0.014)^{***}}{0.09}$$	$$\underset{ (0.013)^{***}}{0.12}$$	$$\underset{(0.014)^{***}}{ 0.05}$$	$$\underset{(0.016)^{***}}{0.14}$$	$$\underset{ (0.016)^{***}}{0.08}$$
$$\gamma _{rR}$$	$$\underset{(0.031)^{***}}{0.08}$$	$$\underset{ (0.010)^{**}}{0.02}$$			$$\underset{(0.030)^{***}}{ 0.11}$$
SBT	$\underset{[0.24]}{1.16}$	$\underset{[0.76]}{0.32}$	$\underset{ [0.31]}{1.01}$	$\underset{[0.29]}{1.06}$	$\underset{[0.17]}{1.39}$
$${\textit{lnL}}$$	$${-8483.63}$$	$${-7339.96}$$	$${-7400.55}$$	$$ {-7433.72}$$	$${-7148.60}$$
*Panel B. Realized measure: m-DAP-HEAVY-R*					
$$\begin{array}{c}(1-\beta _{R}L)(\sigma _{Rt}^{2})^{\frac{\delta _{R}}{2}}=\omega _{R}+(\alpha _{RR}+\gamma _{RR}s_{t-1})L({\textit{RM}}_{t})^{\frac{\delta _{R}}{2} }+\gamma _{Rr}s_{t-1}L(r_{t}^{2})^{\frac{\delta _{r}}{2}} \\ +\phi _{R}{\textit{EPUvol}}_{t-1}+\lambda _{R}{\textit{VIX}}_{t-1}+\zeta _{R}{\textit{BO}}_{t-1}+\vartheta _{R}{\textit{CO}}_{t-1}+\eta _{R}{\textit{ID}}_{t-1}\end{array}$$					
$$\beta _{R}$$	$$\underset{(0.032)^{***}}{0.57}$$	$$\underset{ (0.023)^{***}}{0.73}$$	$$\underset{(0.029)^{***}}{ 0.52}$$	$$\underset{(0.027)^{***}}{0.59}$$	$$\underset{ (0.024)^{***}}{0.60}$$
$$\alpha _{RR}$$	$$\underset{(0.021)^{***}}{0.30}$$	$$\underset{ (0.018)^{***}}{0.19}$$	$$\underset{(0.024)^{***}}{ 0.40}$$	$$\underset{(0.020)^{***}}{0.32}$$	$$\underset{ (0.019)^{***}}{0.32}$$
$$\gamma _{RR}$$		$$\underset{(0.009)^{***}}{0.03}$$			
$$\gamma _{Rr}$$	$$\underset{(0.004)^{***}}{0.05}$$	$$\underset{ (0.004)^{***}}{0.02}$$	$$\underset{(0.005)^{***}}{ 0.05}$$	$$\underset{(0.005)^{***}}{0.04}$$	$$\underset{ (0.004)^{***}}{0.05}$$
$$\phi _{R}$$	$$\underset{(0.007)^{***}}{0.02}$$	$$\underset{ (0.003)^{***}}{0.01}$$		$$\underset{(0.004)^{**}}{0.01} $$	$$\underset{(0.003)^{***}}{0.01}$$
$$\lambda _{R}$$	$$\underset{(0.023)^{**}}{0.05}$$				$$ \underset{(0.011)^{*}}{0.02}$$
$$\zeta _{R}$$	$$\underset{{\textit{MOVE}}}{\underset{(0.024)^{***}}{0.06}}$$	$$\underset{{\textit{BAA}}\_{\textit{AAA}}}{\underset{(0.004)^{***}}{0.01}}$$	$$ \underset{{\textit{BAA}}\_{\textit{AAA}}}{\underset{(0.007)^{***}}{0.02}}$$	$$\underset{{\textit{BAA}}\_{\textit{AAA}}}{\underset{(0.007)^{***}}{0.02}}$$	$$\underset{{\textit{MOVE}}}{ \underset{(0.012)^{***}}{0.05}}$$
$$\vartheta _{R}$$	$$\underset{{\textit{GSCI}}}{\underset{(0.007)^{**}}{0.02}}$$	$$\underset{{\textit{GSCI}}\_{\textit{OIL}}}{\underset{(0.003)^{***}}{0.01}}$$	$$ \underset{{\textit{GSCI}}}{\underset{(0.013)^{***}}{0.04}}$$	$$\underset{{\textit{GSCI}}\_{\textit{OIL}}}{\underset{(0.008)^{***}}{0.04}}$$	$$\underset{{\textit{GSCI}}}{ \underset{(0.007)^{**}}{0.02}}$$
$$\eta _{R}$$	$$\underset{{\textit{ID}}\_{\textit{EMV}}}{\underset{(0.009)^{**}}{0.02}}$$			$$\underset{{\textit{ID}}\_{\textit{EMV}}}{\underset{(0.007)^{***}}{0.03}}$$	$$ \underset{{\textit{ID}}\_{\textit{EMV}}}{\underset{(0.004)^{***}}{0.02}}$$
SBT	$\underset{[0.82]}{0.22}$	$\underset{[0.19]}{1.25}$	$\underset{ [0.34]}{0.96}$	$\underset{[0.47]}{0.73}$	$\underset{[0.62]}{0.51}$
$${\textit{lnL}}$$	$${-7440.84}$$	$${-5600.16}$$	$${-7060.85}$$	$$ {-6853.60}$$	$${-6365.51}$$
*Panel C. Powers* $\delta _{i}$					
$\delta _{r}$	1.40	1.60	1.30	1.40	1.30
$\delta _{R}$	1.20	1.00	1.10	1.10	1.10

The table reports the estimation results of the m-DAP-HEAVY model for each stock index. m-DAP-HEAVY-*r* is the returns equation (Panel A) and m-DAP-HEAVY-*R* is the realized measure equation (Panel B). The estimated powers ($$\delta _{i}$$ ) of returns ($$\delta _{r}$$) and realized measure ($$\delta _{R}$$) are reported in Panel C. SBT denotes the Sign Bias test of Engle and Ng (1993). $${\textit{lnL}}$$ denotes the log-Likelihood value for each specification. The numbers in parentheses are robust standard errors. $$^{***}$$, $$^{**}$$, $$^{*}$$ denote significance at the 0.01, 0.05, 0.10 level, respectively. The numbers in square brackets are p-values. BRAZIL stands for Brazil’s Bovespa index, MEXICO for Mexico’s IPC index, CHINA for the Shanghai Composite index, INDIA for India’s Nifty 50 index, and KOREA for South Korea’s KOSPI index. The m-DAP-HEAVY-*R* equation includes five macro-effects proxied by the following variables: the volatility of US Economic Policy Uncertainty, $${{\textit{EPUvol}}}_{t}$$, the US financial uncertainty (the S&P 500 implied volatility), $${{\textit{VIX}}}_{t}$$, the Bonds effect, $${{\textit{BO}}}_{t}$$, proxied by the Merrill Lynch MOVE index ($${{\textit{MOVE}}}$$) or the Moody’s BAA over AAA corporate bonds spreads ($${{\textit{BAA}}\_{\textit{AAA}}}$$), alternatively, the Commodities effect, $${{\textit{CO}}}_{t}$$, proxied by the S&P GSCI all commodities index ($${{\textit{GSCI}}}$$) or the S&P GSCI oil index ($${{\textit{GSCI}}\_{\textit{OIL}}}$$), alternatively, and the infectious disease effect ($${{\textit{ID}}}_{t}$$) on stock markets captured by the Infectious Disease Equity Market Volatility Tracker ($${{\textit{ID}}\_{\textit{EMV}}}$$). See also the realized variance DAP specification without macro-factors, DAP-HEAVY- *R*, in the Appendix, Table 9

### Macro-effects discussion

From an economic point of view, the macro-effects on stock volatility observed through the m-DAP framework confirm prior studies on the upward volatility trajectory during economic downturns. This counter-cyclical behavior has been mainly shown by the negative sign effect of economic activity leading or coincident indicators with a monthly or quarterly frequency (Engle and Rangel 2008). Turning to the high-frequency domain of the macro-financial linkages, the monthly activity variables should be replaced by possible daily proxies of economic activity to be included as explanatory variables in the realized variance equation. Given the non-negativity restriction, we could not use, among others, the daily term spread, a reliable predictor of GDP (Estrella and Hardouvelis 1991) and significant in the monthly context as evidenced by Conrad and Loch (2015). Based on the rich empirical evidence of the adverse uncertainty effects on economic activity (Caggiano et al. 2017; Colombo 2013; Jones and Olson 2013), we select the daily EPU index to associate stock market volatility with a variable directly linked to economic activity contractive forces. The positive sign consistently estimated here across all specifications for the EPUvol variable is in accordance with prior findings on the positive sign given to macroeconomic uncertainty (Schwert 1989) and unemployment, and the negative sign of the real GDP, industrial production, and consumer sentiment growth (Conrad and Loch 2015). All forces associated with a positive real economic impact exert a negative influence on stock market fluctuations, while the depressive forces exacerbate volatility and are estimated with a positive sign irrespective of the specification chosen by different scholars. Therefore, it is economically plausible for both uncertainty proxies to drive financial volatility higher, at the same time weakening the prevailing macroeconomic conditions.

Against this backdrop, we also selected the sovereign bond yield volatility (or, alternately, the corporate bond default spread level) to identify the credit channel effect on stock markets. Increased volatility in the sovereign bond market (Engle and Rangel 2008) or corporate debt spreads are reasonably correlated with macroeconomic turbulence since they increase the cost of financing for firms and investors and, consequently, reduce activity. Accordingly, the global bond factor parameters are consistently estimated with positive signs across all stock market volatility models (see also Asgharian et al. 2013). Further, the commodity index or, alternately, the oil subindex are included as a volatility determinant, which is found positive and highly significant in all cases. Motivated by the widespread discussion and empirical evidence about the commodity price effects on the macroeconomy in Kilian’s research works (see, for example, Barsky and Kilian 2004), we complement the volatility macro-determinants literature by enriching the set of significant macro-variables for the volatility pattern with commodities and observe the destabilizing impact of higher daily commodity prices, mostly associated with economic downturns, on stock market realized variance. Increased commodity costs for firms’ production supplies impair economic activity and exacerbate equities’ volatility. Finally, we demonstrate that the infectious disease effect on US equities has a detrimental impact exacerbating emerging markets turbulence.

Hence, apart from contributing to the emerging markets realized variance modeling research through the asymmetric, power, and macro-augmentation of the benchmark HEAVY specification applied on emerging economies, we also contribute to the economic sources of volatility by exploring the macro-financial linkages in the high-frequency domain with daily macro-proxies. All daily economic variables that exacerbate developing stock market volatility are associated with weak economic conditions: higher uncertainty, tighter credit conditions, increased commodity prices, and significant disease news impact on equity markets. Moreover, we bridge the macro-finance literature with the high-frequency volatility studies by using the sole economic uncertainty index computed daily. The daily US EPU is applied in the present emerging markets study to reveal the uncertainty spillovers from the US across emerging market economies in Asia and the Americas. The US-led spillover is crucial given its direct connection to the turbulence that surrounds the policy initiatives under Trump’s administration on trade relations or Covid-19 spread, for instance, and the expected governance by the recent new President-elect, which trigger agents’ uncertainty feelings spread over the whole world.

### The crisis effect on realized volatility

After investigating the significant macro-financial linkages in emerging economies, we further explore the significant effect of two crisis events on equity markets, one financial and one health crisis: the 2008 Global Financial Crisis (GFC) and the Covid-19 pandemic period (COVID). The current pandemic has already ignited a new and probably deeper global socio-economic crisis with massive fiscal and monetary stimulus provided by governments, by far larger than the response to the 2008 crisis (Snower 2020). Market turbulence is already observed through markedly increased volatilities close to the peak reached during the 2008 global crisis. Markets are seriously affected by the generalized fear about controversial economic policies to support societies and the financial system, especially in the case of the heavily criticized US government’s delayed and deficient response. Given an unprecedented and challenging threat, namely the rapidly contagious virus across the whole universe, economic agents feel uncertainty about future government policy choices, their implementation, and their potential impact, as well. Even if governments reassure them that the harmful effects of Covid-19 are manageable, skepticism, criticism, and loss of confidence are still there and captured by soaring uncertainty index levels.

Stock market volatility reached a record peak in mid-March when the World Health Organization (WHO) characterized the Coronavirus outbreak as a pandemic while daily EPU levels jumped and still remain in higher territories than during the pre-Covid era. In this vein, we assess the GFC and COVID effect on the daily macro-financial linkages explored in this study by enriching the m-DAP-HEAVY-*R* equation [see Eq. (2)] with the crisis slope dummies ($D_{{\textit{CRISIS}},t}$) on each Heavy, Arch, and Macro parameter [see Eq. (3)], capturing the two crises impact. Based on the Bank for International Settlements (BIS) and WHO timelines the two crisis subsamples are defined as follows:

- GFC: 09/08/2007–31/03/2009. The GFC period starts with the announcement that three major BNP Paribas investment funds are suspended and ends in the first quarter of 2009 with gradual restoration of markets’ ‘tranquillity’.
- COVID: 09/01/2020–30/11/2020. The COVID period starts with the first death reported by China in January 2020 while the pandemic crisis is still in place until the end of our sample.

Following the GFC and COVID timelines, we first construct the respective crisis dummies $D_{{\textit{CRISIS}},t}$, with ${\textit{CRISIS}}={\textit{GFC}},{\textit{COVID}}$, as follows:

- $D_{{\textit{GFC}},t}=1$, if *t* in the GFC period else $D_{{\textit{GFC}},t}=0$
- $D_{{\textit{COVID}},t}=1$, if *t* in the COVID period else $D_{{\textit{COVID}},t}=0$.

Second, we multiply the crisis dummies with the m-DAP-HEAVY-*R* equation’s variables to construct the slope dummies for the respective Heavy, Arch, and Macro effect. The realized variance equation with the crisis impact is estimated as follows:

3 $$\begin{aligned} (1-\beta _{R}L)(\sigma _{Rt}^{2})^{\frac{\delta _{R}}{2}}&=\omega _{R}+[\alpha _{RR}+\alpha _{RR}^{{\textit{CRISIS}}}D_{{\textit{CRISIS}},t-1}+(\gamma _{RR}\nonumber \\&\quad +\gamma _{RR}^{{\textit{CRISIS}}}D_{{\textit{CRISIS}},t-1})s_{t-1}]L({\textit{RM}}_{t})^{\frac{\delta _{R}}{2}} \nonumber \\&\quad +(\gamma _{Rr}+\gamma _{Rr}^{{\textit{CRISIS}}}D_{{\textit{CRISIS}},t-1})s_{t-1}L(r_{t}^{2})^{ \frac{\delta _{r}}{2}}\nonumber \\&\quad +(\phi _{R} +\phi _{R}^{{\textit{CRISIS}}}D_{{\textit{CRISIS}},t-1}){\textit{EPUvol}}_{t-1}\nonumber \\&\quad +(\lambda _{R}\!+\!\lambda _{R}^{{\textit{CRISIS}}}D_{{\textit{CRISIS}},t-1}){\textit{VIX}}_{t-1}\!+\!(\zeta _{R}\!+\!\zeta _{R}^{{\textit{CRISIS}}}D_{{\textit{CRISIS}},t-1}){\textit{BO}}_{t-1} \nonumber \\&\quad +(\vartheta _{R}+\vartheta _{R}^{{\textit{CRISIS}}}D_{{\textit{CRISIS}},t-1}){\textit{CO}}_{t-1}\nonumber \\&\quad +(\eta _{R}+\eta _{R}^{{\textit{CRISIS}}}D_{{\textit{CRISIS}},t-1}){\textit{ID}}_{t-1}, \end{aligned}$$

where the superscript $^{{\textit{CRISIS}}}$ denotes the coefficients of the crisis slope dummies.

Table 4 summarizes the financial and health crisis effect as estimated through alternative restricted forms of Eq. (3) by including separately each crisis slope dummy of the Heavy, Arch, and Macro parameters. The GFC and COVID impacts (Table 4, Panel A and B, respectively) magnify most Heavy terms ($\alpha _{RR}^{{\textit{GFC}}}$, $\alpha _{RR}^{{\textit{COVID}}}$) and Arch asymmetries ($\gamma _{Rr}^{{\textit{GFC}}}$, $\gamma _{Rr}^{{\textit{COVID}}}$). Additional results with both crises dummies jointly significant in the bivariate m-DAP system are reported in Table 10 of the Appendix, where we present the whole equations’ estimations with the preferred combination of GFC and COVID dummies incorporated in the returns and realized measure specifications. The log-Likelihood score of the m-DAP-HEAVY model with crisis dummies demonstrates a slight improvement of the model’s in-sample fit (compare Tables 3 and 10) which is not transferred to the out-of-sample forecasting performance (see Sect. 6). Turning to the crisis impact on the macro-drivers of realized variance, we observe that both GFC- and Covid-induced turbulence in equity markets is important with an inflating impact on the positive effect of the Macro parameters, similarly to the crisis increment estimated for the Heavy and Arch terms. Interestingly, the EPU crisis coefficient ($\phi _{R}^{{\textit{GFC}}}$, $\phi _{R}^{{\textit{COVID}}}$) is always significant, even in the Chinese case, which is estimated insignificant for the whole sample (Table 3, Panel B). Financial uncertainty during GFC and COVID ($\lambda _{R}^{{\textit{GFC}}}$, $\lambda _{R}^{{\textit{COVID}}}$) is estimated for Brazil and Korea only and remains insignificant in the Korean case during GFC. The credit conditions proxies ($\zeta _{R}^{{\textit{GFC}}}$, $\zeta _{R}^{{\textit{COVID}}}$) are also amplified in crisis periods for all indices whereas the commodity factors ($\vartheta _{R}^{{\textit{CRISIS}}}$) become insignificant during the current pandemic in most emerging markets (except for Korea where the crisis effect on commodity regressors is insignificant for both GFC and COVID). The lack of a commodity effect during COVID is rather expected given the sharp drop of crude oil prices following the Covid-19 outbreak (the West Texas Intermediate-WTI crude oil price fell to negative territory instantly in April 2020 for the first time in history), which was partly rebounded with a moderate increase after April 2020 (see also Fig. 4). The GSCI indices remained non-negative during the drop phase and therefore they were preferred compared to alternative commodity variables such as the WTI crude oil price. Finally, the infectious disease devastating news impact is more important during COVID, as expected, while in the GFC subsample, a period closer to the H1N1 pandemic started from the US, the ID_EMV crisis dummy is significant only for India.

Overall, the financial and health crisis detrimental impact on the realized variance is demonstrated through the positive increments added to the variance parameters by the slope dummies included in Eq. (3). Along this line, we, hereby, show once more the counter-cyclical volatility pattern given that the macro-factors associated with weaker economic stance (higher uncertainty, tighter credit, elevated commodity prices, and heavier infectious disease news impact) exacerbate volatility with an upshot intensified during crisis periods.

**Table 4:** The Crisis effect on the Heavy, Arch, and Macro parameters of the m-DAP-HEAVY-*R* equation

	BRAZIL	MEXICO	CHINA	INDIA	KOREA
$$\begin{array}{l}(1-\beta _{R}L)(\sigma _{Rt}^{2})^{\frac{\delta _{R}}{2}}=\omega _{R}+[\alpha _{RR}+\alpha _{RR}^{{\textit{CRISIS}}}D_{{\textit{CRISIS}},t-1}+(\gamma _{RR}+\gamma _{RR}^{{\textit{CRISIS}}}D_{{\textit{CRISIS}},t-1})s_{t-1}]L({\textit{RM}}_{t})^{\frac{\delta _{R}}{2}} \\ +(\gamma _{Rr}+\gamma _{Rr}^{{\textit{CRISIS}}}D_{{\textit{CRISIS}},t-1})s_{t-1}L(r_{t}^{2})^{ \frac{\delta _{r}}{2}}+(\phi _{R}+\phi _{R}^{{\textit{CRISIS}}}D_{{\textit{CRISIS}},t-1}){\textit{EPUvol}}_{t-1} \\ +(\lambda _{R}+\lambda _{R}^{{\textit{CRISIS}}}D_{{\textit{CRISIS}},t-1}){\textit{VIX}}_{t-1}+(\zeta _{R}+\zeta _{R}^{{\textit{CRISIS}}}D_{{\textit{CRISIS}},t-1}){\textit{BO}}_{t-1} \\ +(\vartheta _{R}+\vartheta _{R}^{{\textit{CRISIS}}}D_{{\textit{CRISIS}},t-1}){\textit{CO}}_{t-1}+(\eta _{R}+\eta _{R}^{{\textit{CRISIS}}}D_{{\textit{CRISIS}},t-1}){\textit{ID}}_{t-1}\end{array}$$					
*Panel A: The GFC effect,* ${{\textit{CRISIS}}={\textit{GFC}}}$					
$$\alpha _{RR}^{{\textit{GFC}}}$$	$$\underset{(0.002)^{***}}{0.02}$$	$$ \underset{(0.006)^{**}}{0.01}$$	$$\underset{(0.010)^{**}}{ 0.02}$$	$$\underset{(0.010)^{*}}{0.02}$$	$$\underset{(0.009)^{**} }{0.02}$$
$$\gamma _{RR}^{{\textit{GFC}}}$$		$$\underset{(0.011)^{***}}{0.03}$$			
$$\gamma _{Rr}^{{\textit{GFC}}}$$	$$\underset{(0.011)^{***}}{0.04}$$	$$ \underset{(0.005)^{***}}{0.02}$$	$$\underset{(0.012)^{***}}{0.06}$$	$$\underset{(0.014)^{***}}{0.06}$$	$$\underset{ (0.008)^{***}}{0.03}$$
$$\phi _{R}^{{\textit{GFC}}}$$	$$\underset{(0.035)^{**}}{0.07}$$	$$\underset{ (0.019)^{**}}{0.04}$$	$$\underset{(0.058)^{**}}{0.13}$$	$$ \underset{(0.004)^{**}}{0.01}$$	$$\underset{(0.006)^{***} }{0.02}$$
$$\lambda _{R}^{{\textit{GFC}}}$$	$$\underset{(0.005)^{**}}{0.01}$$				$$ \underset{(0.006)^{{}}}{0.001}$$
$$\zeta _{R}^{{\textit{GFC}}}$$	$$\underset{{\textit{MOVE}}}{\underset{(0.003)^{***}}{ 0.01}}$$	$$\underset{{\textit{BAA}}\_{\textit{AAA}}}{\underset{(0.004)^{***}}{0.01}}$$	$$\underset{{\textit{BAA}}\_{\textit{AAA}}}{\underset{(0.009)^{**}}{0.02}}$$	$$\underset{{\textit{BAA}}\_{\textit{AAA}}}{\underset{(0.008)^{***}}{0.03}}$$	$$\underset{{\textit{MOVE}}}{ \underset{(0.003)^{***}}{0.01}}$$
$$\vartheta _{R}^{{\textit{GFC}}}$$	$$\underset{{\textit{GSCI}}}{\underset{(0.001)^{*}}{0.002}}$$	$$\underset{{\textit{GSCI}}\_{\textit{OIL}}}{\underset{(0.001)^{***}}{0.004}}$$	$$ \underset{{\textit{GSCI}}}{\underset{(0.004)^{***}}{0.01}}$$	$$\underset{{\textit{GSCI}}\_{\textit{OIL}}}{\underset{(0.004)^{***}}{0.01}}$$	$$\underset{{\textit{GSCI}}}{ \underset{(0.002)^{{}}}{0.001}}$$
$$\eta _{R}^{{\textit{GFC}}}$$	$$\underset{{\textit{ID}}\_{\textit{EMV}}}{\underset{(0.009)^{{}}}{0.01}}$$			$$\underset{{\textit{ID}}\_{\textit{EMV}}}{\underset{(0.019)^{**}}{0.04}}$$	$$\underset{{\textit{ID}}\_{\textit{EMV}}}{\underset{(0.083)^{{}}}{0.04}}$$
*Panel B: The COVID effect,* ${{\textit{CRISIS}}={\textit{COVID}}}$					
$$\alpha _{RR}^{{\textit{COVID}}}$$	$$\underset{(0.004)^{***}}{0.02}$$	$$ \underset{(0.009)^{{}}}{0.01}$$	$$\underset{(0.005)^{**}}{0.01}$$	$$ \underset{(0.024)^{{}}}{0.01}$$	$$\underset{(0.005)^{***}}{0.02}$$
$\gamma _{RR}^{{\textit{COVID}}}$		$\underset{(0.013)^{{}}}{0.01}$			
$$\gamma _{Rr}^{{\textit{COVID}}}$$	$$\underset{(0.010)^{***}}{0.04}$$	$$ \underset{(0.006)^{*}}{0.01}$$	$$\underset{(0.016)^{**}}{0.03}$$	$$\underset{(0.022)^{***}}{0.06}$$	$$\underset{(0.016)^{***}}{0.05}$$
$$\phi _{R}^{{\textit{COVID}}}$$	$$\underset{(0.002)^{***}}{0.01}$$	$$ \underset{(0.015)^{**}}{0.03}$$	$$\underset{(0.006)^{***} }{0.06}$$	$$\underset{(0.004)^{**}}{0.01}$$	$$\underset{ (0.030)^{***}}{0.08}$$
$$\lambda _{R}^{{\textit{COVID}}}$$	$$\underset{(0.002)^{***}}{0.01}$$				$$\underset{(0.010)^{***}}{0.03}$$
$$\zeta _{R}^{{\textit{COVID}}}$$	$$\underset{{\textit{MOVE}}}{\underset{(0.005)^{**}}{0.01} }$$	$$\underset{{\textit{BAA}}\_{\textit{AAA}}}{\underset{(0.006)^{*}}{0.01}}$$	$$\underset{{\textit{BAA}}\_{\textit{AAA}}}{\underset{(0.005)^{**}}{0.01}}$$	$$\underset{{\textit{BAA}}\_{\textit{AAA}}}{ \underset{(0.003)^{***}}{0.01}}$$	$$\underset{{\textit{MOVE}}}{\underset{ (0.006)^{**}}{0.01}}$$
$$\vartheta _{R}^{{\textit{COVID}}}$$	$$\underset{{\textit{GSCI}}}{\underset{(0.002)^{{}}}{0.001}}$$	$$\underset{{\textit{GSCI}}\_{\textit{OIL}}}{\underset{(0.004)^{{}}}{0.004}}$$	$$\underset{{\textit{GSCI}}}{ \underset{(0.002)^{{}}}{0.001}}$$	$$\underset{{\textit{GSCI}}\_{\textit{OIL}}}{\underset{ (0.003)^{{}}}{0.001}}$$	$$\underset{{\textit{GSCI}}}{\underset{(0.003)^{***} }{0.01}}$$
$$\eta _{R}^{{\textit{COVID}}}$$	$$\underset{{\textit{ID}}\_{\textit{EMV}}}{\underset{(0.002)^{***} }{0.02}}$$			$$\underset{{\textit{ID}}\_{\textit{EMV}}}{\underset{(0.007)^{***}}{ 0.02}}$$	$$\underset{{\textit{ID}}\_{\textit{EMV}}}{\underset{(0.005)^{***}}{0.02}}$$

The table reports the Crisis effect on the Heavy, Arch, and Macro parameters of the m-DAP-HEAVY-*R* equation. We estimate Eq. (3) including each crisis slope dummy separately and present their estimated coefficients for each stock index realized variance. The slope dummies are calculated by multiplying the Heavy, Arch, and Macro variables with the crisis dummies ($D_{{\textit{CRISIS}},t}$) and the corresponding coefficients are denoted with the superscript ${{\textit{CRISIS}}={\textit{GFC}},{\textit{COVID}}}$, for the Global Financial Crisis (Panel A) and the Covid-19 pandemic period (Panel B), respectively. BRAZIL stands for Brazil’s Bovespa index, MEXICO for Mexico’s IPC index, CHINA for the Shanghai Composite index, INDIA for India’s Nifty 50 index, and KOREA for South Korea’s KOSPI index. The m-DAP-HEAVY-*R* equation includes five macro-effects proxied by the following variables: the volatility of US Economic Policy Uncertainty, ${{\textit{EPUvol}}}_{t}$, the US financial uncertainty (the S&P 500 implied volatility), ${{\textit{VIX}}}_{t}$, the Bonds effect, ${{\textit{BO}}}_{t}$, proxied by the Merrill Lynch MOVE index (${{\textit{MOVE}}}$) or the Moody’s BAA over AAA corporate bonds spreads (${{\textit{BAA}}\_{\textit{AAA}}}$), alternatively, the Commodities effect, ${{\textit{CO}}}_{t}$, proxied by the S&P GSCI all commodities index (${{\textit{GSCI}}}$) or the S&P GSCI oil index (${{\textit{GSCI}}\_{\textit{OIL}}}$), alternatively, and the infectious disease effect (${{\textit{ID}}}_{t}$) on stock markets captured by the Infectious Disease Equity Market Volatility Tracker (${{\textit{ID}}\_{\textit{EMV}}}$). See also the m-DAP bivariate system estimation with the jointly significant crisis dummies in the returns and realized measure equations in the Appendix, Table 10

## Forecasting performance

Beyond demonstrating the in-sample superiority of the m-DAP extension compared to the benchmark model, we investigate the out-of-sample performance of the augmented specification. From a utilitarian point of view, the success of our model can only be claimed through the strong evidence of its superior predictive power. Therefore, we calculate multistep-ahead out-of-sample forecasts in order to compare the forecasting accuracy of our proposed specification with the benchmark model of Shephard and Sheppard (2010) for both returns and realized variance, and the three standard models: the GARCH(1, 1) for returns and the common ARFIMA(1, *d*, 1) and HAR-RV specification for realized variance.

We compute 1-, 5-, 10-, 20-, and 100-step-ahead variance forecasts for the benchmark HEAVY, the DAP, its macro-augmented extension, the m-DAP with crisis dummies (see Table 10, in the Appendix), and the standard models [GARCH(1, 1), ARFIMA(1, *d*, 1) and HAR-RV]. We apply a rolling window in-sample estimation using 3000 observations (the initial in-sample estimation period for BRAZIL spans from 3/1/2000 until 7/3/2012). Each model is re-estimated daily based on a 3000-day rolling sample. The resulted out-of-sample forecasts of each specification calculated for BRAZIL are as follows: 2146 one-step-ahead, 2142 five-step-ahead, 2137 ten-step-ahead, 2127 twenty-step-ahead, and 2047 one-hundred-step-ahead forecasted variances. We then use the time series of the forecasted values to compute the mean square error (MSE) and the QLIKE Loss Function (Patton 2011) of each point forecast compared to the respective actual value. For each formulation and each forecast horizon, we calculate the average MSE and QLIKE to build the ratio of the forecast losses for each extended HEAVY specification (DAP and m-DAP) and each standard model (GARCH, ARFIMA, HAR) to the loss of the benchmark one. A ratio lower than the unity indicates the forecasting superiority of the extended models relative to the benchmark one. A ratio higher than the unity indicates the forecasting superiority of the benchmark model relative to the standard ones. The lowest ratio means the lowest forecast losses, that is the model with the best forecasting performance. Based on the MSE calculations, we further apply the test for the pairwise comparison of competing models (here the benchmark specification vs. the DAP extensions) suggested by Harvey et al. (1998), HLN thereafter. The HLN forecast encompassing test was introduced as a modification to the Diebold-Mariano test (Diebold and Mariano 1995) to account for the fact that models are nested (here the DAP nests the benchmark specification). HLN test whether the differences between the two formulations’ forecasts are statistically significant and the larger model’s forecast losses are lower than the nested model’s ones (see also Clark and McCracken 2001).

We apply the optimal predictor $\left| {\mathbf {r}}_{t}\right| ^{\wedge \varvec{\delta }}$ (see also the optimal predictors derivation in Section B.3, Proposition 3 of the Supplementary Appendix) and calculate the out-of-sample forecasts. The results, presented in Tables 5 and 6 for Brazil’s Bovespa index (similar forecasting results for the other four indices available upon request), clearly show the preference for the macro-augmented extensions over the benchmark models across all time horizons. The m-DAP specification dominates the benchmark model with the lowest MSE and QLIKE (Table 5). For the returns equations (see Table 5, Panel A), the m-DAP formulation dominates the alternative benchmark HEAVY-*r* with the lowest MSE and QLIKE in all forecasting periods and the five- and ten-day forecast losses slightly lower for the m-DAP specification with crisis dummies. In the realized measure equation (see Table 5, Panel B), we obtain the best forecasting performance in the m-DAP specification without crisis dummies in most cases. Comparing the forecast losses of the macro-augmented models with and without crisis dummies, we observe that the differences are small, similarly to the slight differences of their in-sample fit (log-Likelihood scores in Tables 3, 4, and 10). Given the HLN test, the Asymmetric Power formulations perform significantly better than the benchmark models. HLN test results (Table 6) reject the null hypothesis of equal forecasts in favor of the DAP models’ lower forecast losses at 5% significance level while the difference of the forecast losses between the m-DAP specifications with and without crisis dummies is not significant for both returns and realized measure (p-values $>0.100$).

Overall, the extended specifications perform better than the benchmark HEAVY and standard models in the short- and long-term horizons, with the forecasts significantly closer to the actual values for the enriched formulations. Our enhanced in-sample estimations with asymmetries, leverage, and macro-effects have transferred their predictive superiority to the out-of-sample computations. Investors and risk managers should utilize our macro-informed framework’s short-term predictions. At the same time, policymakers can benefit from our superior longer-term forecasts to build reliable scenarios on future financial volatility given the important informational contribution of the daily macro-effects.

**Table 5:** MSE and QLIKE of m-step-ahead out-of-sample forecasts for BRAZIL as a ratio of the benchmark HEAVY model

Specifications$\downarrow$ m-steps $\rightarrow$	MSE					QLIKE				
	1	5	10	20	100	1	5	10	20	100
*Panel A: Stock returns*										
GARCH(1, 1)	1.070	1.161	1.118	1.156	1.211	1.103	1.174	1.161	1.208	1.226
Benchmark HEAVY-*r*	1.000	1.000	1.000	1.000	1.000	1.000	1.000	1.000	1.000	1.000
m-DAP-HEAVY-*r*	$\mathbf {0.692}$	0.874	0.911	$\mathbf { 0.935}$	$\mathbf {0.949}$	$\mathbf {0.651}$	0.833	0.896	$\mathbf {0.873}$	$\mathbf {0.952}$
m-DAP-HEAVY-*r*-crisis	0.694	$\mathbf {0.870}$	$\mathbf {0.906}$	0.945	0.961	0.655	$\mathbf {0.829}$	$\mathbf { 0.894}$	0.878	0.953
*Panel B: Realized measure*										
ARFIMA(1, *d*, 1)	1.052	1.099	1.171	1.165	1.203	1.066	1.085	1.168	1.169	1.228
HAR-RV	1.031	1.086	1.163	1.127	1.188	1.043	1.071	1.147	1.151	1.198
Benchmark HEAVY-*R*	1.000	1.000	1.000	1.000	1.000	1.000	1.000	1.000	1.000	1.000
DAP-HEAVY-*R*	0.711	0.832	0.894	0.782	0.867	0.690	0.751	0.799	0.764	0.808
m-DAP-HEAVY-*R*	$\mathbf {0.693}$	$\mathbf {0.825}$	$\mathbf { 0.875}$	0.776	$\mathbf {0.860}$	$\mathbf {0.678}$	$\mathbf {0.739}$	$\mathbf {0.780}$	$\mathbf {0.741}$	$\mathbf {0.802}$
m-DAP-HEAVY-*R*-crisis	0.699	0.826	0.879	$\mathbf {0.770}$	0.858	0.681	0.743	0.781	0.740	0.806

The table reports the mean square error (MSE) and QLIKE of m-step-ahead out-of-sample forecasts for BRAZIL as a ratio of the Benchmark HEAVY model. We first compute the 1-, 5-, 10-, 20-, and 100-step-ahead variance forecasts of returns (for each returns competing model: GARCH( 1, 1), Benchmark HEAVY-*r*, m-DAP-HEAVY-*r*, and m-DAP-HEAVY-*r* with crisis dummies) and realized variance (for each realized variance competing model: ARFIMA(1, *d*, 1), HAR-RV, Benchmark HEAVY- *R*, DAP-HEAVY-*R*, m-DAP-HEAVY-*R*, and m-DAP-HEAVY-*R* with crisis dummies) with a rolling window procedure (3000 observations for in-sample estimation, initial in-sample period: 3/1/2000–7/3/2012). The average MSE and QLIKE of the time series of forecasted values versus the actuals are calculated for each forecast horizon and each model. The ratio is built by dividing the two loss functions (MSE and QLIKE) of each formulation over the benchmark HEAVY losses. A ratio lower than the unity indicates the forecasting superiority of the respective model relative to the benchmark one. The lowest ratio means the lowest forecast losses, that is the model with the best forecasting performance. Bold numbers indicate minimum values across the different specifications

**Table 6:** HLN Forecast encompassing test results for BRAZIL (p-values)

Specifications$\downarrow$ m-steps$\rightarrow$	1	5	10	20	100
*Panel A: Stock returns* (*HEAVY*-*r*)					
Benchmark vs. m-DAP	0.004	0.025	0.034	0.050	0.049
Benchmark vs. m-DAP-crisis	0.008	0.021	0.030	0.056	0.053
m-DAP vs. m-DAP-crisis	0.256	0.589	0.888	0.754	0.432
*Panel B: Realized measure* (*HEAVY*-*R*)					
Benchmark vs. DAP	0.025	0.027	0.046	0.041	0.051
Benchmark vs. m-DAP	0.006	0.016	0.039	0.040	0.045
Benchmark vs. m-DAP-crisis	0.005	0.018	0.042	0.043	0.044
m-DAP vs. m-DAP-crisis	0.238	0.411	0.657	0.698	0.626

The table reports the p-values of the Harvey et al. (1998) test (HLN). The HLN forecast encompassing test indicates whether the differences between two nested models’ forecasts are statistically significant. The test’s null hypothesis of equal forecasting performance against the one-sided alternative that the extended outperforms the nested specification is rejected when we observe low p-values ($$<0.100$$). All DAP models (extended) outperform the benchmark (nested) ones (p-values $$ <0.056$$). The comparison of the m-DAP and the m-DAP with crisis dummies shows that the difference between their respective forecasts is not statistically significant (p-values $${>0.100}$$)

## The indirect uncertainty effect

Following the estimation of the benchmark HEAVY system with asymmetries, power transformations, and macroeconomic effects, and its sensitivity to financial and health crises, we investigate the drastic influence of uncertainty on financial volatility. Over the decade following the global turmoil, which sharply sparked the interest in the role of uncertainty and the relevant research increasingly gained momentum following an accelerating pace, the most widespread metrics documented, or proxies used, have referred to macroeconomic, financial, and policy uncertainty. They all share a common and highly plausible stylized fact: their guiding significance with a detrimental impact on the health of the economy and financial markets, which is stage-contingent (dampening economic activity with higher magnitude in shakier times—see also our crisis sensitivity analysis in Sect. 5.4). Despite the rapidly growing EPU literature, it appears that the empirical work on the realized volatility dynamics driven by EPU is limited, with evidence still scant for the emerging world, in particular. Consequently, the present study fills a notable gap in the extant EPU literature. We elucidate whether EPU exerts considerable influence on the HEAVY volatility modeling framework and on specific parameters of the macro-augmented asymmetric power specification. Our work differs from the existing literature in the use of the daily EPU index as a daily realized volatility determinant in emerging stock markets, with major implications for macro-informed trading in financial markets and policymakers’ financial stability concerns and systemic risk oversight. Obviously, the particular EPU-volatility link has not yet been thoroughly assessed.

### The indirect EPU impact on realized volatility

Against this backdrop, we have already highlighted the direct positive effect, in line with Pastor and Veronesi (2013), and the forecasting power of daily EPUvol on realized volatility within the m-DAP framework in Sects. 5 and 6. In this Section, we extend our empirical analysis by focusing more specifically on the first volatility macro-determinant of the realized measure equation, that is the economic uncertainty impact on stock indices realized variance. In what follows, we prove the significant EPU effect on the Heavy, Arch, uncertainty, bonds, commodities, and infectious disease news impact on the stock market realized variance. The m-DAP realized volatility equation is estimated using eight restricted forms alternately to examine each EPU effect separately with the following interaction terms: (i)–(iii) $\alpha _{RR}^{{\textit{EPU}}}$, $\gamma _{RR}^{{\textit{EPU}}}$, and $\gamma _{Rr}^{{\textit{EPU}}}$, are the parameters of the lagged EPU multiplied by the lagged realized variance and the two asymmetric effects, capturing the EPU effect on the Heavy ($\alpha _{RR}$ and $\gamma _{RR}$) and asymmetric Arch ($\gamma _{Rr}$) parameters, (iv)–(viii) $\phi _{R}^{{\textit{EPU}}}$, $\lambda _{R}^{{\textit{EPU}}}$ , $\zeta _{R}^{{\textit{EPU}}}$, $\vartheta _{R}^{{\textit{EPU}}}$, and $\eta _{R}^{{\textit{EPU}}}$ measure the EPU effect on EPUvol, financial uncertainty, bonds, commodities, and disease news proxies, respectively. The interaction terms are again calculated through the multiplication of the log-transformed EPU index level by the respective variable and included in Eq. (2) as follows:

4 $$\begin{aligned} (1-\beta _{R}L)(\sigma _{Rt}^{2})^{\frac{\delta _{R}}{2}}&=\omega _{R}+[\alpha _{RR}+\alpha _{RR}^{{\textit{EPU}}}{\textit{EPU}}_{t-1}+(\gamma _{RR}+\gamma _{RR}^{{\textit{EPU}}}{\textit{EPU}}_{t-1})s_{t-1}]L({\textit{RM}}_{t})^{\frac{\delta _{R}}{2}} \nonumber \\&\quad +(\gamma _{Rr}+\gamma _{Rr}^{{\textit{EPU}}}{\textit{EPU}}_{t-1})s_{t-1}L(r_{t}^{2})^{\frac{ \delta _{r}}{2}}\nonumber \\&\quad +(\phi _{R}+\phi _{R}^{{\textit{EPU}}}{\textit{EPU}}_{t-1}){\textit{EPUvol}}_{t-1}+(\lambda _{R}+\lambda _{R}^{{\textit{EPU}}}{\textit{EPU}}_{t-1}){\textit{VIX}}_{t-1} \nonumber \\&\quad +(\zeta _{R}+\zeta _{R}^{{\textit{EPU}}}{\textit{EPU}}_{t-1}){\textit{BO}}_{t-1}\nonumber \\&\quad +(\vartheta _{R}+\vartheta _{R}^{{\textit{EPU}}}{\textit{EPU}}_{t-1}){\textit{CO}}_{t-1}+(\eta _{R}+\eta _{R}^{{\textit{EPU}}}{\textit{EPU}}_{t-1}){\textit{ID}}_{t-1}, \end{aligned}$$

where the superscript $^{{\textit{EPU}}}$ denotes the coefficients of the EPU interaction terms.

The direct EPUvol effect is already apparent through the significant $\phi _{R}$ estimated in the m-DAP-HEAVY-*R* equation (Sect. 5.2, Table 3, Panel B). Table 7 summarizes the indirect EPU effects on realized volatility of the five emerging stock indices. We present the uncertainty impact on each parameter given by the alternative restricted forms of Eq. (4), including each interaction term one by one. They are all estimated with highly significant positive signed coefficients, signifying the amplifying EPU impact on each parameter. Intriguingly, within the macro-enriched DAP specification, we demonstrate that higher economic policy uncertainty means a stronger influence of EPU volatility, financial uncertainty, credit conditions, commodity market benchmarks, and infectious disease news on the realized measure. It is noticeable that EPU absorbs a significant part of the Heavy, Arch, and Macro-effects. Within the uncertainty literature, the link between credit condition tightening and uncertainty has recently been investigated by Alessandri and Mumtaz (2019), who associate the rising financing costs for firms with credit market uncertainty, while the commodities-uncertainty relation is widely explored by Antonakakis et al. (2014), Aloui et al. (2016), and Fang et al. (2018) among others. Most notably, Antonakakis et al. (2017) focus on the oil prices-stock market volatility link. According to our review of the flourishing research on uncertainty effects, academics have not yet covered the EPU, credit, commodities, and disease macro-effects on intra-daily emerging markets’ financial volatility and the EPU amplifying role on the credit and production cost channel, alongside the pandemic news impact, as well, which is plainly visible here through the HEAVY framework.

**Table 7:** The EPU effect on the Heavy, Arch, and Macro parameters of the m-DAP-HEAVY-*R* equation

	BRAZIL	MEXICO	CHINA	INDIA	KOREA
$$\begin{array}{l}(1-\beta _{R}L)(\sigma _{Rt}^{2})^{\frac{\delta _{R}}{2}}=\omega _{R}+[\alpha _{RR}+\alpha _{RR}^{{\textit{EPU}}}{\textit{EPU}}_{t-1}+(\gamma _{RR}+\gamma _{RR}^{{\textit{EPU}}}{\textit{EPU}}_{t-1})s_{t-1}]L({\textit{RM}}_{t})^{\frac{\delta _{R}}{2}} \\ +(\gamma _{Rr}+\gamma _{Rr}^{{\textit{EPU}}}{\textit{EPU}}_{t-1})s_{t-1}L(r_{t}^{2})^{\frac{ \delta _{r}}{2}}+(\phi _{R}+\phi _{R}^{{\textit{EPU}}}{\textit{EPU}}_{t-1}){\textit{EPUvol}}_{t-1}+(\lambda _{R}+\lambda _{R}^{{\textit{EPU}}}{\textit{EPU}}_{t-1}){\textit{VIX}}_{t-1} \\ +(\zeta _{R}+\zeta _{R}^{{\textit{EPU}}}{\textit{EPU}}_{t-1}){\textit{BO}}_{t-1}+(\vartheta _{R}+\vartheta _{R}^{{\textit{EPU}}}{\textit{EPU}}_{t-1}){\textit{CO}}_{t-1}+(\eta _{R}+\eta _{R}^{{\textit{EPU}}}{\textit{EPU}}_{t-1}){\textit{ID}}_{t-1}\end{array}$$					
$$\alpha _{RR}^{{\textit{EPU}}}$$	$$\underset{(0.010)^{***}}{0.12}$$	$$ \underset{(0.005)^{***}}{0.05}$$	$$\underset{(0.011)^{***}}{0.16}$$	$$\underset{(0.005)^{***}}{0.11}$$	$$\underset{ (0.009)^{***}}{0.12}$$
$$\gamma _{RR}^{{\textit{EPU}}}$$		$$\underset{(0.004)^{***}}{0.01}$$			
$$\gamma _{Rr}^{{\textit{EPU}}}$$	$$\underset{(0.002)^{***}}{0.03}$$	$$ \underset{(0.002)^{***}}{0.01}$$	$$\underset{(0.002)^{***}}{0.02}$$	$$\underset{(0.003)^{***}}{0.02}$$	$$\underset{ (0.002)^{***}}{0.02}$$
$$\phi _{R}^{{\textit{EPU}}}$$	$$\underset{(0.004)^{***}}{0.01}$$	$$\underset{(0.002)^{***}}{0.01}$$		$$\underset{(0.002)^{***}}{0.01}$$	$$\underset{(0.002)^{***}}{0.01}$$
$$\lambda _{R}^{EPU}$$	$$\underset{(0.005)^{**}}{0.01}$$				$$ \underset{(0.003)^{**}}{0.01}$$
$$\zeta _{R}^{{\textit{EPU}}}$$	$$\underset{{\textit{MOVE}}}{\underset{(0.004)^{***}}{ 0.01}}$$	$$\underset{{\textit{BAA}}\_{\textit{AAA}}}{\underset{(0.001)^{***}}{0.01}}$$	$$\underset{{\textit{BAA}}\_{\textit{AAA}}}{\underset{(0.003)^{***}}{0.01}}$$	$$ \underset{{\textit{BAA}}\_{\textit{AAA}}}{\underset{(0.004)^{**}}{0.01}}$$	$$\underset{{\textit{MOVE}} }{\underset{(0.002)^{***}}{0.01}}$$
$$\vartheta _{R}^{{\textit{EPU}}}$$	$$\underset{{\textit{GSCI}}}{\underset{(0.002)^{***} }{0.02}}$$	$$\underset{{\textit{GSCI}}\_{\textit{OIL}}}{\underset{(0.001)^{***}}{0.01}} $$	$$\underset{{\textit{GSCI}}}{\underset{(0.006)^{***}}{0.02}}$$	$$ \underset{{\textit{GSCI}}\_{\textit{OIL}}}{\underset{(0.002)^{***}}{0.01}}$$	$$ \underset{{\textit{GSCI}}}{\underset{(0.004)^{**}}{0.01}}$$
$$\eta _{R}^{{\textit{EPU}}}$$	$$\underset{{\textit{ID}}\_{\textit{EMV}}}{\underset{(0.004)^{*}}{0.01}}$$			$$\underset{{\textit{ID}}\_{\textit{EMV}}}{\underset{(0.003)^{***}}{0.01}}$$	$$ \underset{{\textit{ID}}\_{\textit{EMV}}}{\underset{(0.001)^{***}}{0.01}}$$

The table reports the indirect EPU effect on the Heavy, Arch, and Macro parameters of the m-DAP-HEAVY-*R* equation. We estimate Eq. (4) including each EPU interaction term separately and present their estimated coefficients for each stock index realized variance. The interaction terms are calculated by multiplying the Heavy, Arch, and Macro variables with the EPU log-level (${{\textit{EPU}}}_{t}$) and the corresponding coefficients are denoted with the superscript EPU. BRAZIL stands for Brazil’s Bovespa index, MEXICO for Mexico’s IPC index, CHINA for the Shanghai Composite index, INDIA for India’s Nifty 50 index, and KOREA for South Korea’s KOSPI index. The m-DAP-HEAVY-*R* equation includes five macro-effects proxied by the following variables: the volatility of US Economic Policy Uncertainty, ${{\textit{EPUvol}}} _{t}$, the US financial uncertainty (the S&P 500 implied volatility), ${{\textit{VIX}}}_{t}$, the Bonds effect, ${{\textit{BO}}}_{t}$, proxied by the Merrill Lynch MOVE index (${{\textit{MOVE}}}$) or the Moody’s BAA over AAA corporate bonds spreads (${{\textit{BAA}}\_{\textit{AAA}}}$), alternatively, the Commodities effect, ${{\textit{CO}}}_{t}$, proxied by the S&P GSCI all commodities index (${{\textit{GSCI}}}$) or the S&P GSCI oil index (${{\textit{GSCI}}\_{\textit{OIL}}}$), alternatively, and the infectious disease effect (${{\textit{ID}}}_{t}$) on stock markets captured by the Infectious Disease Equity Market Volatility Tracker (${{\textit{ID}}\_{\textit{EMV}}}$)

### The indirect EPU impact on realized volatility during crisis

Next, we combine the EPU with the crisis impact to estimate the uncertainty effect on each realized variance parameter during crisis periods, separately. The in-crisis EPU impact on emerging equity realized volatility dynamics is captured by the coefficients with the superscript $^{{\textit{EPU}}\_{\textit{CR}}}$ in the following equation:

5 $$\begin{aligned} (1-\beta _{R}L)(\sigma _{Rt}^{2})^{\frac{\delta _{R}}{2}}&=\omega _{R}+[\alpha _{RR}+\alpha _{RR}^{{\textit{EPU}}\_{\textit{CR}}}D_{{\textit{CRISIS}},t-1}{\textit{EPU}}_{t-1}\nonumber \\&\quad +(\gamma _{RR}+\gamma _{RR}^{{\textit{EPU}}\_{\textit{CR}}}D_{{\textit{CRISIS}},t-1}{\textit{EPU}}_{t-1})s_{t-1}]L({\textit{RM}}_{t})^{\frac{\delta _{R}}{ 2}}\nonumber \\&\quad +(\gamma _{Rr}+\gamma _{Rr}^{{\textit{EPU}}\_{\textit{CR}}}D_{{\textit{CRISIS}},t-1}{\textit{EPU}}_{t-1})s_{t-1}L(r_{t}^{2})^{\frac{\delta _{r} }{2}}\nonumber \\&\quad +(\phi _{R}+\phi _{R}^{{\textit{EPU}}\_{\textit{CR}}}D_{{\textit{CRISIS}},t-1}{\textit{EPU}}_{t-1}){\textit{EPUvol}}_{t-1}\nonumber \\&\quad +(\lambda _{R}+\lambda _{R}^{{\textit{EPU}}\_{\textit{CR}}}D_{{\textit{CRISIS}},t-1}{\textit{EPU}}_{t-1}){\textit{VIX}}_{t-1}\nonumber \\&\quad +(\zeta _{R}+\zeta _{R}^{{\textit{EPU}}\_{\textit{CR}}}D_{{\textit{CRISIS}},t-1}{\textit{EPU}}_{t-1}){\textit{BO}}_{t-1}\nonumber \\&\quad +(\vartheta _{R}+\vartheta _{R}^{{\textit{EPU}}\_{\textit{CR}}}D_{{\textit{CRISIS}},t-1}{\textit{EPU}}_{t-1}){\textit{CO}}_{t-1}\nonumber \\&\quad +(\eta _{R}+\eta _{R}^{{\textit{EPU}}\_CR}D_{{\textit{CRISIS}},t-1}{\textit{EPU}}_{t-1}){\textit{ID}}_{t-1}. \end{aligned}$$

where ${\textit{CRISIS}}$ and ${\textit{CR}}={\textit{GFC}},{\textit{COVID}}$. Each EPU interaction term of Eq. (4) is multiplied with the crisis slope dummies applied in Eq. (3) in order to identify the indirect EPU effect during crises.

Table 8 reports the crisis impact on the EPU interaction terms as estimated through restricted forms of Eq. (5) by including each crisis-EPU term separately.^5^ Similar to our crisis analysis (Table 4), we observe that the EPU interaction terms are significantly inflated during the 2008 financial turmoil (Panel A) in most cases. The Heavy, Arch, and EPUvol effects are augmented through the uncertainty (level) channel in the GFC period for all indices included, and the Chinese market as well, contrary to the EPUvol impact whose EPU interaction term is estimated insignificant and excluded in the whole sample for China (see Table 7, $\phi _{Rr}^{{\textit{EPU}}}$ insignificant for the Chinese index). US financial uncertainty, credit, and commodity conditions are also intensified for the models where they are incorporated apart from the Korean case. The indirect EPU effect on infectious disease news is important only for India in the GFC period while, during COVID (Panel B), it is significant for Brazil, India, and Korea. Furthermore, in the COVID period, most Heavy, credit, and commodity factors do not receive a statistically significant EPU impact, whereas Arch asymmetries and EPUvol interaction terms escalate the respective effect across all markets.

**Table 8:** The EPU effect during crisis on the Heavy, Arch, and Macro parameters of the m-DAP-HEAVY-*R* equation

	BRAZIL	MEXICO	CHINA	INDIA	KOREA
$$\begin{array}{l}(1-\beta _{R}L)(\sigma _{Rt}^{2})^{\frac{\delta _{R}}{2}}=\omega _{R}+[\alpha _{RR}+\alpha _{RR}^{{\textit{EPU}}\_{\textit{CR}}}D_{{\textit{CRISIS}},t-1}{\textit{EPU}}_{t-1} \\ +(\gamma _{RR}+\gamma _{RR}^{{\textit{EPU}}\_{\textit{CR}}}D_{{\textit{CRISIS}},t-1}{\textit{EPU}}_{t-1})s_{t-1}]L({\textit{RM}}_{t})^{\frac{\delta _{R}}{ 2}}+(\gamma _{Rr}+\gamma _{Rr}^{{\textit{EPU}}\_{\textit{CR}}}D_{{\textit{CRISIS}},t-1}{\textit{EPU}}_{t-1})s_{t-1}L(r_{t}^{2})^{\frac{\delta _{r} }{2}} \\ +(\phi _{R}+\phi _{R}^{{\textit{EPU}}\_{\textit{CR}}}D_{{\textit{CRISIS}},t-1}{\textit{EPU}}_{t-1}){\textit{EPUvol}}_{t-1}+(\lambda _{R}+\lambda _{R}^{{\textit{EPU}}\_{\textit{CR}}}D_{{\textit{CRISIS}},t-1}{\textit{EPU}}_{t-1}){\textit{VIX}}_{t-1} \\ +(\zeta _{R}+\zeta _{R}^{{\textit{EPU}}\_{\textit{CR}}}D_{{\textit{CRISIS}},t-1}{\textit{EPU}}_{t-1}){\textit{BO}}_{t-1}+(\vartheta _{R}+\vartheta _{R}^{{\textit{EPU}}\_{\textit{CR}}}D_{{\textit{CRISIS}},t-1}{\textit{EPU}}_{t-1}){\textit{CO}}_{t-1} \\ +(\eta _{R}+\eta _{R}^{{\textit{EPU}}\_{\textit{CR}}}D_{{\textit{CRISIS}},t-1}{\textit{EPU}}_{t-1}){\textit{ID}}_{t-1}\end{array}$$					
*Panel A: The EPU effect during the GFC period*, ${{\textit{CRISIS}},{\textit{CR}}={\textit{GFC}}}$					
$$\alpha _{RR}^{{\textit{EPU}}\_{\textit{GFC}}}$$	$$\underset{(0.004)^{***}}{0.01}$$	$$ \underset{(0.003)^{**}}{0.01}$$	$$\underset{(0.004)^{*}}{0.01}$$	$$\underset{(0.007)^{**}}{0.01}$$	$$\underset{(0.004)^{*}}{0.01} $$
$$\gamma _{RR}^{{\textit{EPU}}\_{\textit{GFC}}}$$		$$\underset{(0.005)^{***}}{0.02}$$			
$$\gamma _{Rr}^{{\textit{EPU}}\_{\textit{GFC}}}$$	$$\underset{(0.006)^{***}}{0.02}$$	$$ \underset{(0.003)^{***}}{0.01}$$	$$\underset{(0.006)^{***}}{0.03}$$	$$\underset{(0.007)^{***}}{0.03}$$	$$\underset{ (0.004)^{***}}{0.02}$$
$$\phi _{R}^{{\textit{EPU}}\_{\textit{GFC}}}$$	$$\underset{(0.023)^{*}}{0.04}$$	$$\underset{ (0.010)^{**}}{0.02}$$	$$\underset{(0.029)^{**}}{0.06}$$	$$ \underset{(0.004)^{**}}{0.01}$$	$$\underset{(0.007)^{***} }{0.02}$$
$$\lambda _{R}^{{\textit{EPU}}\_{\textit{GFC}}}$$	$$\underset{(0.005)^{**}}{0.01}$$				$$\underset{(0.003)^{{}}}{0.003}$$
$$\zeta _{R}^{{\textit{EPU}}\_{\textit{GFC}}}$$	$$\underset{{\textit{MOVE}}}{\underset{(0.004)^{**}}{ 0.01}}$$	$$\underset{{\textit{BAA}}\_{\textit{AAA}}}{\underset{(0.002)^{***}}{0.01}}$$	$$\underset{{\textit{BAA}}\_{\textit{AAA}}}{\underset{(0.005)^{**}}{0.01}}$$	$$\underset{{\textit{BAA}}\_{\textit{AAA}}}{\underset{(0.005)^{***}}{0.02}}$$	$$\underset{{\textit{MOVE}}}{ \underset{(0.002)^{{}}}{0.001}}$$
$$\vartheta _{R}^{{\textit{EPU}}\_{\textit{GFC}}}$$	$$\underset{{\textit{GSCI}}}{\underset{(0.002)^{*}}{ 0.003}}$$	$$\underset{{\textit{GSCI}}\_{\textit{OIL}}}{\underset{(0.001)^{**}}{0.002}}$$	$$ \underset{{\textit{GSCI}}}{\underset{(0.002)^{***}}{0.01}}$$	$$\underset{ {\textit{GSCI}}\_{\textit{OIL}}}{\underset{(0.002)^{***}}{0.01}}$$	$$\underset{{\textit{GSCI}}}{ \underset{(0.001)^{{}}}{0.001}}$$
$$\eta _{R}^{{\textit{EPU}}\_{\textit{GFC}}}$$	$$\underset{{\textit{ID}}\_{\textit{EMV}}}{\underset{(0.086)^{{}}}{0.02}}$$			$$\underset{{\textit{ID}}\_{\textit{EMV}}}{\underset{(0.009)^{**}}{0.02}}$$	$$ \underset{{\textit{ID}}\_{\textit{EMV}}}{\underset{(0.065)^{{}}}{0.05}}$$
*Panel B: The EPU effect during the COVID period*, ${{\textit{CRISIS}},{\textit{CR}}={\textit{COVID}}}$					
$\alpha _{RR}^{{\textit{EPU}}\_{\textit{COVID}}}$	$\underset{(0.009)^{{}}}{0.01}$	$\underset{ (0.003)^{{}}}{0.002}$	$\underset{(0.002)^{*}}{0.003}$	$\underset{ (0.011)^{{}}}{0.01}$	$\underset{(0.007)^{{}}}{0.003}$
$\gamma _{RR}^{{\textit{EPU}}\_{\textit{COVID}}}$		$\underset{(0.006)^{{}}}{0.004}$			
$$\gamma _{Rr}^{{\textit{EPU}}\_{\textit{COVID}}}$$	$$\underset{(0.007)^{***}}{0.02}$$	$$\underset{(0.003)^{*}}{0.004}$$	$$\underset{(0.007)^{*}}{0.01}$$	$$ \underset{(0.011)^{**}}{0.03}$$	$$\underset{(0.007)^{***} }{0.02}$$
$$\phi _{R}^{{\textit{EPU}}\_{\textit{COVID}}}$$	$$\underset{(0.002)^{**}}{0.004}$$	$$ \underset{(0.009)^{**}}{0.02}$$	$$\underset{(0.005)^{***} }{0.02}$$	$$\underset{(0.004)^{**}}{0.01}$$	$$\underset{ (0.010)^{***}}{0.03}$$
$$\lambda _{R}^{{\textit{EPU}}\_{\textit{COVID}}}$$	$$\underset{(0.002)^{**}}{0.004}$$				$$\underset{(0.004)^{***}}{0.01}$$
$$\zeta _{R}^{{\textit{EPU}}\_{\textit{COVID}}}$$	$$\underset{{\textit{MOVE}}}{\underset{(0.008)^{{}}}{0.001}}$$	$$\underset{{\textit{BAA}}\_{\textit{AAA}}}{\underset{(0.001)^{*}}{0.002}}$$	$$\underset{{\textit{BAA}}\_{\textit{AAA}}}{\underset{(0.004)^{{}}}{0.003}}$$	$$\underset{{\textit{BAA}}\_{\textit{AAA}}}{\underset{ (0.007)^{{}}}{0.004}}$$	$$\underset{{\textit{MOVE}}}{\underset{(0.003)^{**}}{ 0.01}}$$
$$\vartheta _{R}^{{\textit{EPU}}\_{\textit{COVID}}}$$	$$\underset{{\textit{GSCI}}}{\underset{(0.004)^{{}}}{ 0.001}}$$	$$\underset{{\textit{GSCI}}\_{\textit{OIL}}}{\underset{(0.002)^{{}}}{0.002}}$$	$$ \underset{{\textit{GSCI}}}{\underset{(0.001)^{{}}}{0.001}}$$	$$\underset{{\textit{GSCI}}\_{\textit{OIL}}}{ \underset{(0.002)^{{}}}{0.001}}$$	$$\underset{{\textit{GSCI}}}{\underset{(0.002)^{**}}{0.004}}$$
$$\eta _{R}^{{\textit{EPU}}\_{\textit{COVID}}}$$	$$\underset{{\textit{ID}}\_{\textit{EMV}}}{\underset{(0.003)^{***}}{0.01}}$$			$$\underset{{\textit{ID}}\_{\textit{EMV}}}{\underset{(0.003)^{***}}{0.01}}$$	$$\underset{{\textit{ID}}\_{\textit{EMV}}}{\underset{(0.002)^{***}}{ 0.01}}$$

The table reports the indirect EPU effect during the two CRISIS periods (Global Financial Crisis, GFC—Panel A and Covid-19 pandemic period, COVID—Panel B) on the Heavy, Arch, and Macro parameters of the m-DAP-HEAVY-*R* equation. We estimate Eq. (5) including each crisis slope dummy for the EPU interaction terms separately and present their estimated coefficients for each stock index realized variance. The slope dummies are calculated by multiplying the EPU interaction terms of the Heavy, Arch, and Macro variables with the crisis dummies ($D_{{\textit{CRISIS}},t},{{\textit{CRISIS}}={\textit{GFC}},{\textit{COVID}}}$) and the corresponding coefficients are denoted with the superscript ${{\textit{EPU}}\_{\textit{CR}}={\textit{EPU}}\_{\textit{GFC}},{\textit{EPU}}\_{\textit{COVID}}}$, for the GFC and COVID subsamples, respectively. BRAZIL stands for Brazil’s Bovespa index, MEXICO for Mexico’s IPC index, CHINA for the Shanghai Composite index, INDIA for India’s Nifty 50 index, and KOREA for South Korea’s KOSPI index. The m-DAP-HEAVY-*R* equation includes five macro-effects proxied by the following variables: the volatility of US Economic Policy Uncertainty, ${{\textit{EPUvol}}} _{t}$, the US financial uncertainty (the S&P 500 implied volatility), ${{\textit{VIX}}}_{t}$, the Bonds effect, ${{\textit{BO}}}_{t}$, proxied by the Merrill Lynch MOVE index (${{\textit{MOVE}}}$) or the Moody’s BAA over AAA corporate bonds spreads (${{\textit{BAA}}\_{\textit{AAA}}}$), alternatively, the Commodities effect, ${{\textit{CO}}}_{t}$, proxied by the S&P GSCI all commodities index (${{\textit{GSCI}}}$)or the S&P GSCI oil index (${{\textit{GSCI}}\_{\textit{OIL}}}$), alternatively, and the infectious disease effect (${{\textit{ID}}}_{t}$) on stock markets captured by the Infectious Disease Equity Market Volatility Tracker (${{\textit{ID}}\_{\textit{EMV}}}$)

All in all, our contribution to the EPU literature consists of the new empirical evidence we provide on the positive link between daily EPU and emerging markets realized volatility and the US EPU volatility spillovers across emerging economies which are sensitive to crisis periods and higher EPU levels. Within the HEAVY framework, we firstly demonstrate the US EPUvol destabilizing impact on emerging stock markets with financial volatility investigated in a daily frequency. Secondly, we show that the leverage and heavy effects on realized variance are considerably magnified in financial and health crisis events and under higher prevailing uncertainty conditions. Thirdly, and most interestingly from an economic perspective, the increased VIX and volatility in credit conditions (or higher credit risk pricing in cases where the Moody’s corporate default spreads are applied), the rising prices in commodities, the disease news overflow, all three phenomena associated with economic downturns, exacerbate realized volatility to a degree intensified by elevated US EPU and crisis turbulence. Finally, we complement the literature on EPU spillovers (see, for example, Gabauer and Gupta 2018; Balli et al. 2017, and Klößner and Sekkel 2014) by providing evidence of the daily uncertainty spillover effects from the US to emerging stock markets’ intra-daily volatility. We have demonstrated that policy uncertainty in a specific country is not confined to the country’s borders but is propagated across the whole world immediately (only the first EPU lag is examined in this study).

## Policy implications discussion

Nowadays, our results should urge policymakers to consider and closely investigate the side effects of US policy uncertainty generated in recent years mostly by Trump’s controversial rhetoric and administration for the whole developing world. Overall, we demonstrate that emerging financial markets in Asia and the Americas are destabilized by higher policy uncertainty in the US economy directly (EPU volatility) and indirectly (EPU log-level), besides US financial uncertainty, global commodity and credit market conditions, and the infectious disease news impact, as well. The macro-effects on index volatility are significantly inflated by elevated EPU levels and the detrimental impact of crisis events, both financial and health emergencies. Turning to the policy implications of the macro-augmented high-frequency volatility model, our findings suggest that policymakers and authorities supervising and regulating the financial system should take into account reliable volatility forecasts in designing macro- and micro-prudential policy responses. Regulators could consider the macro-informed financial volatility forecasts of the m-DAP-HEAVY model across the whole risk management process of the financial system (identification of risk sources, assessment of the nature of risk factors, risk measurement, and risk mitigation) and the financial stability oversight tools, such as early warning systems, macro stress tests on financial institutions, and bank capital frameworks.

For example, based on our sensitivity analysis with the crisis magnifying impact and the EPU spillover effect on volatility macro-determinants, early warning systems for emerging economies should consider the US uncertainty channel and past global turmoil periods in identifying forward-looking signals which could imply a future market crash. Further, the macro stress test scenario inputs, which include, among others, stock market volatility predictions for the financial institutions’ trading books, should consider macro-informed volatility estimates to account for the macro-effects on financial markets. Economic uncertainty in one major country has been shown to play a decisive role across multiple regions’ equities. Accordingly, it is essential for supervisory authorities to add the US uncertainty factor in banks’ stress tests while facing the US policy turbulence. Moreover, complying with the capital and risk frameworks set by supervisors (Basel committee and central banks), financial institutions measure their trading portfolio’s market risk through internal models of daily Value-at-Risk (VaR) in order to estimate the potential trading losses over a pre-defined holding period for a given confidence level and define the corresponding capital charges. The most important input in the VaR calculation is the one-day volatility forecast of each risk factor relevant to the financial instruments under scope. Stock index price volatilities are widely used in the VaR computation of stock portfolios. Thus, reliable macro-informed volatility forecasts, provided by a macro-augmented volatility modeling framework, improve the VaR estimates considerably. Given that the market risk capital requirement is calculated on the trading portfolio’s total 99% VaR (absolute value, 60-day average) adjusted by the penalty of the backtesting exceptions (higher than 4 in the 250-day sample), supervisors should encourage banks to improve their market risk internal models with more accurate macro-informed volatility forecasts which better capture the loss distribution without inflating the capital charges.

Beyond our tangible results’ implications for policymakers, the volatility forecasts produced by the m-DAP-HEAVY model are directly applicable to a wide range of business finance operations. Alongside the well-established risk management practice of the trading VaR estimation, portfolio managers should rely on the proposed framework to predict future volatility in asset allocation and minimum-variance portfolio selection complying with their clients’ risk appetite. Risk-averse investors’ mandates specify low volatility boundaries on their portfolio positions, while risk lovers allow for higher volatilities on the risk-return trade-off of their investments. Accurate volatility predictions can also be used in a forward-looking performance evaluation context, through the risk-adjusted metrics, i.e. the Sharpe or the Treynor risk-adjusted return ratios (see, for example, Ben Ameur et al. 2018). Traders and risk managers focus on the volatility trajectory in derivatives pricing, volatility targeting strategies, and macro-informed trading decisions. Trading and hedging in financial markets depend on risk factors whose predicted volatilities are the main input of any pricing function applied. Lastly, financial chiefs consider volatility forecasts when they decide on investment projects or funding choices (bond and equity valuation defining the cost of capital) given that expected future cash-flow variation is a critical factor in business analytics.

## Conclusions

Our study has examined the HEAVY model and its extension with leverage, power transformations, and macro-characteristics. For the realized measure, our empirical results favor the macro-augmented cross asymmetric power specification, where the lags of both powered variables—squared negative returns, and realized variance—move the dynamics of the power transformed conditional variance of the latter. Similarly, modeling the returns with a double asymmetric power process, we found that not only the powered realized measure asymmetry but the power transformed squared negative returns, as well, help to forecast the conditional variance of the latter. The macro-augmentation of the asymmetric power model ensures the superiority of our contribution, which can be implemented in several investment and risk management practices. We further demonstrated the forecasting dominance of the extended specifications over the benchmark HEAVY and standard volatility models through the out-of-sample forecasting across multiple short- and long-term horizons.

Moreover, we demarcate our study from previous literature by estimating the significant US uncertainty effect on the power of leverage (Heavy and Arch), and the macro-determinants of emerging markets realized variance. The US-led uncertainty spillovers shed light on new evidence for volatility modeling and macro-financial linkages literature. Our findings’ novelty is twofold: Given higher (lower) daily US policy uncertainty levels, mostly associated with economic downturns (upturns), (i) heavy and leverage effects become more (less) acute in realized variance modeling, and (ii) US EPU volatility, financial uncertainty, credit conditions, commodity market benchmarks, and disease news impact on emerging financial volatility increases (decreases). Similarly, financial and health crisis events magnify further the Heavy, Arch, and Macro parameters of the bivariate system and the EPU indirect impact on the volatility drivers, as well.

Our empirical findings on the nexus between low-frequency daily squared returns, high-frequency intra-daily realized measures, and daily macro-proxies provide a volatility forecasting framework with important implications for policymakers and market practitioners, from investors, risk and portfolio managers up to financial chiefs, leaving ample room for future research on further HEAVY model extensions. Therefore, policymakers and market players may use the more general framework to closely track and forecast financial volatility patterns in the process of devising stringent policies, enforcing the financial system’s regulations to preserve financial stability, deciding on asset allocation, hedging strategies, and investment projects. This US-led uncertainty spillover phenomenon, in particular, should be immediately recognized, monitored, and mitigated by regulators amid inconceivable fears stimulated by US politics, such as controversial policy initiatives on trade relations and the recent Covid-19 tragedy, among other critical issues. As part of future research, it would be interesting to extend our study to exchange rate market volatility and several other asset classes using alternative macro-proxies for each type of asset.

### Supplementary Information

Below is the link to the electronic supplementary material.

Supplementary material 1 (pdf 463 KB)

## Appendix

See Appendix Tables 9 and 10.

**Table 9:** The DAP-HEAVY-*R* equation (without macro-financial factors)

$$(1-\beta _{R}L)(\sigma _{Rt}^{2})^{\frac{\delta _{R}}{2} }=\omega _{R}+(\alpha _{RR}+\gamma _{RR}s_{t-1})L({\textit{RM}}_{t})^{\frac{\delta _{R} }{2}}+\gamma _{Rr}s_{t-1}L(r_{t}^{2})^{\frac{\delta _{r}}{2}}$$					
	BRAZIL	MEXICO	CHINA	INDIA	KOREA
$$\beta _{R}$$	$$\underset{(0.028)^{***}}{0.62}$$	$$\underset{ (0.022)^{***}}{0.74}$$	$$\underset{(0.028)^{***}}{ 0.54}$$	$$\underset{(0.024)^{***}}{0.62}$$	$$\underset{ (0.022)^{***}}{0.63}$$
$$\alpha _{RR}$$	$$\underset{(0.021)^{***}}{0.29}$$	$$\underset{ (0.018)^{***}}{0.19}$$	$$\underset{(0.024)^{***}}{ 0.40}$$	$$\underset{(0.021)^{***}}{0.33}$$	$$\underset{ (0.020)^{***}}{0.32}$$
$$\gamma _{RR}$$		$$\underset{(0.008)^{***}}{0.03}$$			
$$\gamma _{Rr}$$	$$\underset{(0.004)^{***}}{0.05}$$	$$\underset{ (0.003)^{***}}{0.02}$$	$$\underset{(0.005)^{***}}{ 0.05}$$	$$\underset{(0.005)^{***}}{0.03}$$	$$\underset{ (0.004)^{***}}{0.04}$$
$${\textit{lnL}}$$	$${-7442.81}$$	$${-5609.61}$$	$$ {-7066.53}$$	$${-6555.89}$$	$${-6372.53}$$
*Powers* $\delta _{i}$					
$\delta _{r}$	1.40	1.60	1.30	1.40	1.30
$\delta _{R}$	1.20	1.00	1.10	1.10	1.10

The table reports the estimation results of the DAP-HEAVY-*R* equation for each stock index realized measure without macro-regressors (Table 3, Panel B reports the corresponding m-DAP-HEAVY-*R* equation with macro-effects). The estimated powers ($$\delta _{i}$$) of returns ($$\delta _{r}$$) and realized measure ($$\delta _{R}$$) are reported in lower Panel. $${\textit{lnL}}$$ denotes the log-Likelihood value for each specification. The numbers in parentheses are robust standard errors. $$^{***}$$, $$^{**}$$, $$^{*}$$ denote significance at the 0.01,  0.05,  0.10 level, respectively. The numbers in square brackets are p-values. BRAZIL stands for Brazil’s Bovespa index, MEXICO for Mexico’s IPC index, CHINA for the Shanghai Composite index, INDIA for India’s Nifty 50 index, and KOREA for South Korea’s KOSPI index

**Table 10:** The m-DAP-HEAVY model with the Crisis effect

	BRAZIL	MEXICO	CHINA	INDIA	KOREA
*Panel A. Stock returns: m-DAP-HEAVY-**r* *with crisis dummies*					
$$\begin{array}{l}(1-\beta _{r}L)(\sigma _{rt}^{2})^{\frac{\delta _{r}}{2}}=\omega _{r}+(\gamma _{rr}+\gamma _{rr}^{{\textit{GFC}}}D_{{\textit{GFC}},t-1})s_{t-1}L(r_{t}^{2})^{\frac{ \delta _{r}}{2}} \\ \quad +[\alpha _{rR}+\alpha _{rR}^{{\textit{COVID}}}D_{{\textit{COVID}},t-1}+(\gamma _{rR}+\gamma _{rR}^{{\textit{COVID}}}D_{{\textit{COVID}},t-1})s_{t-1}]L({\textit{RM}}_{t})^{\frac{\delta _{R}}{2}}\end{array}$$					
$$\beta _{r}$$	$$\underset{(0.040)^{***}}{0.84 }$$	$$\underset{(0.090)^{***}}{0.93}$$	$$\underset{(0.068)^{***}}{0.78}$$	$$\underset{(0.020)^{***}}{0.86}$$	$$ \underset{(0.034)^{***}}{0.77}$$
$$\alpha _{rR}$$	$$\underset{(0.046)^{**}}{0.09}$$		$$\underset{(0.066)^{***}}{0.21}$$	$$\underset{(0.016)^{***}}{0.07}$$	$$\underset{(0.041)^{***}}{0.21}$$
$$\alpha _{rR}^{{\textit{COVID}}}$$	$$\underset{(0.027)^{*}}{0.05 }$$		$$\underset{(0.018)^{**}}{0.04}$$	$$\underset{(0.010)^{**}}{0.02}$$	$$\underset{(0.009)^{**}}{0.02}$$
$$\gamma _{rr}$$	$$\underset{(0.014)^{***}}{ 0.08}$$	$$\underset{(0.011)^{***}}{0.10}$$	$$\underset{ (0.013)^{***}}{0.05}$$	$$\underset{(0.016)^{***}}{ 0.14}$$	$$\underset{(0.017)^{***}}{0.08}$$
$$\gamma _{rr}^{{\textit{GFC}}}$$	$$\underset{(0.011)^{***}}{0.03}$$	$$\underset{(0.020)^{**}}{0.04}$$	$$\underset{ (0.022)^{**}}{0.04}$$	$$\underset{(0.012)^{***}}{0.03}$$	
$$\gamma _{rR}$$	$$\underset{(0.032)^{***}}{ 0.09}$$	$$\underset{(0.010)^{**}}{0.02}$$			$$\underset{ (0.030)^{***}}{0.11}$$
$$\gamma _{rR}^{{\textit{COVID}}}$$		$$\underset{(0.001)^{***}}{0.003}$$			$$\underset{(0.018)^{***}}{0.04}$$
$${\textit{lnL}}$$	$${-8481.33}$$	$${-7338.11}$$	$$ {-7398.45}$$	$${-7432.34}$$	$${-7148.07}$$
*Panel B. Realized measure: m-DAP-HEAVY-**R* *with crisis dummies*					
$$\begin{array}{l} (1-\beta _{R}L)(\sigma _{Rt}^{2})^{\frac{\delta _{R}}{2}}=\omega _{R}+[\alpha _{RR}+\alpha _{RR}^{{\textit{GFC}}}D_{{\textit{GFC}},t-1}\\ \quad +(\gamma _{RR}+\gamma _{RR}^{{\textit{GFC}}}D_{{\textit{GFC}},t-1})s_{t-1}]L({\textit{RM}}_{t})^{\frac{ \delta _{R}}{2}}+(\gamma _{Rr}+\gamma _{Rr}^{{\textit{COVID}}}D_{{\textit{COVID}},t-1})s_{t-1}L(r_{t}^{2})^{\frac{\delta _{r}}{2}}\\ \quad +\phi _{R}{\textit{EPUvol}}_{t-1}+\lambda _{R}{\textit{VIX}}_{t-1}+\zeta _{R}{\textit{BO}}_{t-1}+\vartheta _{R}{\textit{CO}}_{t-1}+\eta _{R}{\textit{ID}}_{t-1}\end{array}$$					
$$\beta _{R}$$	$$\underset{(0.039)^{***}}{0.51 }$$	$$\underset{(0.024)^{***}}{0.73}$$	$$\underset{(0.032)^{***}}{0.50}$$	$$\underset{(0.029)^{***}}{0.59}$$	$$ \underset{(0.027)^{***}}{0.59}$$
$$\alpha _{RR}$$	$$\underset{(0.026)^{***}}{ 0.38}$$	$$\underset{(0.020)^{***}}{0.20}$$	$$\underset{ (0.024)^{***}}{0.45}$$	$$\underset{(0.024)^{***}}{ 0.35}$$	$$\underset{(0.016)^{***}}{0.34}$$
$$\alpha _{RR}^{{\textit{GFC}}}$$	$$\underset{(0.006)^{***}}{0.02}$$		$$\underset{(0.010)^{**}}{0.02}$$	$$\underset{ (0.014)^{**}}{0.03}$$	$$\underset{(0.010)^{**}}{0.02}$$
$$\gamma _{RR}$$		$$\underset{(0.009)^{**}}{0.02 }$$			
$$\gamma _{RR}^{{\textit{GFC}}}$$		$$\underset{(0.011)^{**} }{0.03}$$			
$$\gamma _{Rr}$$	$$\underset{(0.005)^{***}}{ 0.02}$$	$$\underset{(0.002)^{***}}{0.01}$$	$$\underset{ (0.005)^{***}}{0.04}$$	$$\underset{(0.006)^{***}}{ 0.03}$$	$$\underset{(0.004)^{***}}{0.05}$$
$$\gamma _{Rr}^{{\textit{COVID}}}$$	$$\underset{(0.017)^{**}}{0.04}$$	$$\underset{(0.005)^{**}}{0.01}$$	$$\underset{(0.016)^{*}}{0.03}$$	$$\underset{(0.026)^{***}}{0.06}$$	$$\underset{ (0.008)^{***}}{0.03}$$
$$\phi _{R}$$	$$\underset{(0.008)^{***}}{0.02} $$	$$\underset{(0.004)^{***}}{0.01}$$		$$\underset{ (0.004)^{**}}{0.01}$$	$$\underset{(0.003)^{**}}{0.01}$$
$$\lambda _{R}$$	$$\underset{(0.029)^{***}}{ 0.11}$$				$$\underset{(0.018)^{***}}{0.06}$$
$$\zeta _{R}$$	$$\underset{{\textit{MOVE}}}{\underset{(0.028)^{*} }{0.05}}$$	$$\underset{{\textit{BAA}}\_{\textit{AAA}}}{\underset{(0.004)^{**}}{0.01}}$$	$$ \underset{{\textit{BAA}}\_{\textit{AAA}}}{\underset{(0.008)^{***}}{0.02}}$$	$$\underset{{\textit{BAA}}\_{\textit{AAA}}}{\underset{(0.005)^{**}}{0.01}}$$	$$\underset{{\textit{MOVE}}}{ \underset{(0.014)^{**}}{0.03}}$$
$$\vartheta _{R}$$	$$\underset{{\textit{GSCI}}}{\underset{ (0.010)^{**}}{0.02}}$$	$$\underset{{\textit{GSCI}}\_{\textit{OIL}}}{\underset{ (0.002)^{***}}{0.01}}$$	$$\underset{{\textit{GSCI}}}{\underset{ (0.014)^{***}}{0.05}}$$	$$\underset{{\textit{GSCI}}\_{\textit{OIL}}}{\underset{ (0.010)^{***}}{0.05}}$$	$$\underset{{\textit{GSCI}}}{\underset{ (0.008)^{*}}{0.01}}$$
$$\eta _{R}$$	$$\underset{{\textit{ID}}\_{\textit{EMV}}}{\underset{ (0.009)^{**}}{0.02}}$$			$$\underset{{\textit{ID}}\_{\textit{EMV}}}{\underset{ (0.008)^{***}}{0.02}}$$	$$\underset{{\textit{ID}}\_{\textit{EMV}}}{\underset{ (0.005)^{**}}{0.01}}$$
$${\textit{lnL}}$$	$${-7440.66}$$	$${-5600.11}$$	$$ {-7060.04}$$	$${-6853.20}$$	$${-6365.10}$$
*Panel C: Powers* $\delta _{i}$					
$\delta _{r}$	1.40	1.60	1.30	1.40	1.30
$\delta _{R}$	1.20	1.00	1.10	1.10	1.10

The table reports the estimation results of the m-DAP-HEAVY model with crisis dummies for each stock index. m-DAP-HEAVY-*r* with crisis dummies is the returns equation (Panel A) and m-DAP-HEAVY-*R* with crisis dummies is the realized measure equation (Panel B). The estimated powers ($$\delta _{i}$$) of returns ($$\delta _{r}$$) and realized measure ($$\delta _{R}$$) are reported in Panel C. The crisis slope dummies included in each equation arethe ones jointly significant and statistically preferred. The slope dummies are calculated by multiplying the Heavy and Arch variables with the crisis dummies ($$D_{{\textit{CRISIS}},t}$$) and the corresponding coefficients are denoted with the superscript $${{\textit{CRISIS}}={\textit{GFC}},{\textit{COVID}}}$$, for the Global Financial Crisis and the Covid-19 pandemic period, respectively. $${\textit{lnL}}$$ denotes the log-Likelihood value for each specification. The numbers in parentheses are robust standard errors. $$^{***}$$, $$^{**}$$, $$^{*}$$ denote significance at the 0.01,  0.05,  0.10 level, respectively. The numbers in square brackets are p-values. BRAZIL stands for Brazil’s Bovespa index, MEXICO for Mexico’s IPC index, CHINA for the Shanghai Composite index, INDIA for India’s Nifty 50 index, and KOREA for South Korea’s KOSPI index. The m-DAP-HEAVY-*R* with crisis dummies equation includes five macro-effects proxied by the following variables: the volatility of US Economic Policy Uncertainty, $${{\textit{EPUvol}}}_{t}$$, the US financial uncertainty (the S&P 500 implied volatility), $${{\textit{VIX}}}_{t}$$, the Bonds effect, $$ {{\textit{BO}}}_{t}$$, proxied by the Merrill Lynch MOVE index ($${{\textit{MOVE}}}$$) or the Moody’s BAA over AAA corporate bonds spreads ($${{\textit{BAA}}\_{\textit{AAA}}}$$), alternatively, the Commodities effect, $${{\textit{CO}}}_{t}$$, proxied by the S&P GSCI all commodities index ($${{\textit{GSCI}}}$$) or the S&P GSCI oil index ($$ {{\textit{GSCI}}\_{\textit{OIL}}}$$), alternatively, and the infectious disease effect ($${{\textit{ID}}}_{t}$$) on stock markets captured by the Infectious Disease Equity Market Volatility Tracker ($${{\textit{ID}}\_{\textit{EMV}}}$$)

## Footnotes

1 High-frequency-based-volatility (see, Shephard and Sheppard 2010).

2 This type of asymmetry was introduced by Glosten et al. (1993).

3 The log-transformed series are always positive because all series’ values are higher than one. Since the lower bound of our macro-regressors’ series is not one but zero, we, alternatively, included the regressors divided by 100 (EPU, VIX, MOVE, WTI) and 10,000 (GSCI). This resulted in similar estimated coefficients in terms of level and significance within the HEAVY framework (results available upon request).

4 Further research could consider an exponential HEAVY specification to address the non-negativity limitations.

5 The estimation results of the whole Eqs. (3), (4), and (5), when each EPU, crisis, and EPU under crisis effect, is included separately, are omitted due to space considerations. They are available upon request by the authors.

## Declarations

### Availability of data and material

Data available on request from the authors. The data that support the findings of this study are derived from Thomson Reuters Datastream and http://www.policyuncertainty.com/.

### Code availability

OxMetrics 7 is used for the econometric estimations.
