# Supplementary material for: Emerging stock market volatility and economic fundamentals: the importance of US uncertainty spillovers, financial and health crises
Source: Ann Oper Res. 2021 Apr 21;313(2):1077–116. doi: 10.1007/s10479-021-04042-y (PMC8059431; doi:10.1007/s10479-021-04042-y)
Supplement: Supplementary file 1 — Supplementary material 1 (pdf 463 KB) [file 10479_2021_4042_MOESM1_ESM.pdf]

SUPPLEMENTARY APPENDIX to the paper entitled: 'Emerging Stock Market Volatility and Economic Fundamentals: the importance of US Uncertainty Spillovers, Financial and Health Crises.'

M. Karanasos[†], S. Yfanti[‡,*], J. Hunter[†]

[†]*Brunel University London, UK;* [‡]*Loughborough University, UK*

[*]Address for correspondence: Dr Stavroula Yfanti, School of Business and Economics, Loughborough University, Epinal Way, Loughborough, LE11 3TU, UK. Telephone: +44 (0)1509 227091. Email: stavyfan@gmail.com. ORCID ID: 0000-0001-8071-916X

A Notation

Throughout the Appendix, we adhere to the following conventions: ($\mathbb{Z}_{>0}$) $\mathbb{Z}$, and $\mathbb{Z}_{\geq 0}$ stand for the sets of (positive) integers, and non-negative integers respectively. Similarly, ($\mathbb{R}_{>0}$) $\mathbb{R}$ and $\mathbb{R}_{\geq 0}$ stands for the set of (positive) real numbers, and non-negative real numbers respectively. We will use upper (lower) case boldface symbols to refer to square matrices (vectors). That is, $\mathbf{y} = [y_i]_{i=1,\dots,N}$ is an $N \times 1$ column vector, $\mathbf{Y} = [y_{ij}]_{i,j=1,\dots,N}$ is a square matrix of order N (hereafter, we will drop the subscript for notational simplicity), and $diag[\mathbf{y}]$, $dg[\mathbf{Y}]$ denote diagonal matrices with elements y_i and y_{ii}, respectively. Further, using standard notation, $\mathbf{Y}'$ and $\mathbf{Y}^{-1}$ are the transpose and the inverse of the square matrix $\mathbf{Y}$. $\mathbf{Y}^{\wedge k} = [y_{ij}^k]$ is the elementwise exponentiation whereas $\mathbf{y}^{\wedge \mathbf{x}} = [y_i^{x_i}]$, that is the ith entry of vector $\mathbf{y}$ is raised to the ith entry of vector $\mathbf{x}$. Superscripts within parentheses or brackets (e.g., $(.)^{(m)}$) designate the index position of the corresponding term (e.g., m-th term) of a sequence, so as to distinguish position indices from power exponents. Moreover, $\mathbf{Y}^k = \prod_{i=1}^{k} \mathbf{Y}$ means that the matrix $\mathbf{Y}$ is raised to the power of k. The elementwise expectation operator is denoted by $\mathbb{E}$, i.e., $\mathbb{E}(\mathbf{Y}) = [\mathbb{E}(y_{ij})]$ (similarly, $\mathbb{E}(\mathbf{Y} \,|\mathcal{F}_{t-1}\,)$ denotes the elementwise, conditional on time $t-1$, expectation operator). We will refer to the elementwise

absolute value of $\mathbf{Y}$ as $|\mathbf{Y}| = [|y_{ij}|]$. Finally, let $\mathbf{Y}^{\otimes 2} = \mathbf{Y} \otimes \mathbf{Y}$, where $\otimes$ is the Kronecker product of two matrices, and $vec(\mathbf{Y})$ is a vector in which the columns of the matrix $\mathbf{Y}$ are stacked one underneath the other.

B Multivariate System

In this Appendix, we will examine the theoretical properties of the multivariate AP-HEAVY model. We will consider the N-dimensional vector process, $\mathbf{r}_t = [r_{it}]$, $i = 1, \ldots, N$, $N \in \mathbb{Z}_{\geq 1}$, $t \in \mathbb{Z}$. For example, for our bivariate case (see Section 3 in the main body of the paper), $r_{1t} = r_t$, and $r_{2t} = \widetilde{RM}_t$, and the vector $\mathbf{r}_t$ is characterized by the relation

$$\mathbf{r}_t = \mathbf{Z}_t \boldsymbol{\sigma}_t, \qquad \text{(B.1)}$$

where $\mathbf{Z}_t = diag[\mathbf{e}_t]$, $\mathbf{e}_t = [e_{it}]$, and $\boldsymbol{\sigma}_t = [\sigma_{it}]$ is $\mathcal{F}_{t-1}$ measurable with $\mathcal{F}_{t-1} = \sigma(\mathbf{r}_{t-1}, \mathbf{r}_{t-2}, \ldots)$ with $\boldsymbol{\sigma}_t > \mathbf{0}$ for all t. That is, $\mathbf{r}_t = [e_{it}\sigma_{it}]$. Analogously with the assumptions in Section 3 of the paper the stochastic vector $\mathbf{e}_t = [e_{it}]$ is independent and identically distributed $(i.i.d)$.

In the N-dimensional (constant conditional correlation) multivariate GARCH model $\mathbf{e}_t$ has zero mean, unit variance, and positive definite time invariant conditional correlation matrix $\mathbf{R}_e = [\rho_{ij}]$ with $\rho_{ii} = 1$. The conditional covariance matrix of $\mathbf{r}_t$ is denoted by $\mathbf{H}_t = \mathbb{E}(\mathbf{r}_t \mathbf{r}_t' | \mathcal{F}_{t-1})$, and it is given by $\mathbf{H}_t = \boldsymbol{\Sigma}_t \mathbf{R} \boldsymbol{\Sigma}_t$, where $\boldsymbol{\Sigma}_t = diag[\boldsymbol{\sigma}_t] = diag[\mathbf{H}_t^{\frac{1}{2}}]$.

The N-dimensional AP-HEAVY$(1,1)$ model is a generalization of the bivariate specification in eq. (1) of the main paper, and it is given by

$$(\mathbf{I}_N - \mathbf{B}L)\boldsymbol{\sigma}_t^{\wedge \boldsymbol{\delta}} = \boldsymbol{\omega} + L\mathbf{A}_t |\mathbf{r}_t|^{\wedge \boldsymbol{\delta}}, \qquad \text{(B.2)}$$

where $\boldsymbol{\delta} = [\delta_i]$ is the vector with the power parameters with $\delta_i \in \mathbb{R}_{>0}$ for all i, $\boldsymbol{\sigma}_t^{\wedge \boldsymbol{\delta}} = [\sigma_{it}^{\delta_i}]$, and $|\mathbf{r}_t|^{\wedge \boldsymbol{\delta}} = [|e_{it}|^{\delta_i} \sigma_{it}^{\delta_i}]$ (we recall that $\mathbf{r}_t$ and $\boldsymbol{\sigma}_t$ have been defined in eq. (B.1)). $\mathbf{I}_N$ is the N-dimensional identity matrix, $\mathbf{B} = [\beta_{ii}]$ is a diagonal matrix (of order N); $\boldsymbol{\omega} = [\omega_i]$ is a vector that contains the drifts; $\mathbf{A}_t = \mathbf{A} + \boldsymbol{\Gamma}_t$, where $\mathbf{A} = [\alpha_{ij}]$ and $\boldsymbol{\Gamma}_t = [\gamma_{ij} s_{jt}]$, are N-dimensional full matrices. Note that $\boldsymbol{\Gamma}_t$ can be written as $\boldsymbol{\Gamma}_t = \boldsymbol{\Gamma} diag[\mathbf{s}_t]$ where $\boldsymbol{\Gamma} = [\gamma_{ij}]$ and $\mathbf{s}_t = [s_{it}]$. The cross diagonal elements of $\mathbf{A}$ capture the shock (or unconditional) spillovers, whereas those of $\boldsymbol{\Gamma}_t$ capture the asymmetric shock spillovers.

B.1 VARMA Representation

In order to derive the optimal predictors, we need to obtain the weak VARMA representation of the model in eq. (B.2). First, we will introduce the following definitions.

Definition 1 *i) Let* $\mathbf{Z}(\boldsymbol{\delta}) = \mathbb{E}(|\mathbf{Z_t}|^{\wedge \boldsymbol{\delta}})$ *be a diagonal matrix with the element occupying the i-th entry denoted by* $z_i = \mathbb{E}(|e_{it}|^{\delta_i})$*,*

ii) Define the serially uncorrelated vector with zero mean as follows: $\mathbf{v}_t(\boldsymbol{\delta}) = |\mathbf{r}_t|^{\wedge\boldsymbol{\delta}} - \mathbb{E}(|\mathbf{r}_t|^{\wedge\boldsymbol{\delta}} |\mathcal{F}_{t-1})$*. To lighten the notation, in what follows we drop the subscript* $\boldsymbol{\delta}$*. In view of eq. (B.1),* $\mathbf{v}_t$ *can be written as*

$$\mathbf{v}_t = |\mathbf{r}_t|^{\wedge\boldsymbol{\delta}} - \mathbf{Z}\boldsymbol{\sigma}_t^{\wedge\boldsymbol{\delta}} = \left(|\mathbf{Z}_t|^{\wedge\boldsymbol{\delta}} - \mathbf{Z}\right)\boldsymbol{\sigma}_t^{\wedge\boldsymbol{\delta}}.$$

(we recall that $\boldsymbol{\delta}$ is given in eq. (B.2)).

Proposition 1 *The weak VARMA(1,1) representation of the* N*-dimensional AP-HEAVY* $(1,1)$ *process is given by*

$$[\mathbf{I}_N - L\mathbf{C}_t]\boldsymbol{\sigma}_t^{\wedge\boldsymbol{\delta}} = \boldsymbol{\omega} + L\mathbf{A}_t\mathbf{v}_t, \tag{B.3}$$

where

$$\mathbf{C}_t = \mathbf{B} + \mathbf{A}_t\mathbf{Z}$$

($\mathbf{B}$, and $\mathbf{A}_t$ have been defined in eq. (B.2); notice that $\mathbf{C}_t$ depends on $\boldsymbol{\delta}$, but again in order to simplify the notation we will use $\mathbf{C}_t$ instead of, for example, $\mathbf{C}_t(\boldsymbol{\delta})$).

The proof is trivial: we add and subtract $\mathbf{A}_{t-1}\mathbf{Z}\boldsymbol{\sigma}_{t-1}^{\wedge\boldsymbol{\delta}}$ in the right-hand side of eq. (B.2).

Next, let us call

$$\mathbf{D}_{t,k} = \prod_{r=0}^{k-1}\mathbf{C}_{t-1-r}, \tag{B.4}$$

where $k \in \mathbb{Z}_{\geq 1}$. We further extend the definition of $\mathbf{D}_{t,k}$ by assigning the initial matrix value $\mathbf{D}_{t,0} = \mathbf{I}_N$.

B.2 General Solution

Next, we will present the general solution, which generates all the main time series properties of the AP-HEAVY multivariate system.

Theorem 1 *The general solution of the weak VARMA representation in eq. (B.3) under the initial matrix value* $\boldsymbol{\sigma}_{t-k}^{\wedge\boldsymbol{\delta}}$*, is given by*

$$\boldsymbol{\sigma}_t^{\wedge\boldsymbol{\delta}} = \underbrace{\sum_{r=0}^{k-1}\mathbf{D}_{t,r-1}\left(\boldsymbol{\omega} + \mathbf{A}_{t-r}\mathbf{v}_{t-r}\right) +}_{\textit{(Particular Solution)}} \underbrace{\mathbf{D}_{t,k}\boldsymbol{\sigma}_{t-k}^{\wedge\boldsymbol{\delta}}}_{\textit{(Homogeneous Sol.)}}. \tag{B.5}$$

The proof is trivial. It is obtained by using repeated substitution in eq. (B.3).

In the above Proposition $\boldsymbol{\sigma}_t^{\wedge\boldsymbol{\delta}}$ is decomposed into two parts. The homogeneous solution, which consists of the initial (matrix) value $\boldsymbol{\sigma}_{t-k}^{\wedge\boldsymbol{\delta}}$ times $\mathbf{D}_{t,k}$, and the particular one that is formed by products involving the matrix $\mathbf{D}_{t,r-1}$ times i) the drift $\boldsymbol{\omega}$, and ii) the matrix $\mathbf{A}_{t-r}$ times the serially uncorrelated vector $\mathbf{v}_{t-r}$.

Remark 1 *When* $k = 1$ *the general solution in Theorem 1 coincides with eq. (B.3). This is a consequence of the following statement:* $\mathbf{D}_{t,0} = \mathbf{I}_N$ *and* $\mathbf{D}_{t,1} = \mathbf{C}_{t-1}$ *(see eq. (B.4)).*

B.3 Optimal Predictors

In what follows, we will obtain the linear predictor of the AP-HEAVY system.

First, we will introduce some additional notation.

i)Let the expected value of $\mathbf{C}_t$ and $\mathbf{A}_t$ be denoted as $\mathbf{C} = \mathbb{E}(\mathbf{C}_t)$ and $\overline{\mathbf{A}} = \mathbb{E}(\mathbf{A}_t)$ respectively (where $\mathbf{C}_t$ is given in eq. (B.3)). Thus

$$\mathbf{C} = \mathbf{B} + \overline{\mathbf{A}}\mathbf{Z}, \text{ with } \overline{\mathbf{A}} = \left(\mathbf{A} + \mathbf{\Gamma}\frac{\mathbf{1}}{\mathbf{2}}\right) \tag{B.6}$$

(since $\mathbb{E}[diag[\mathbf{s}_t]] = \mathbb{E}[diag[\mathbf{s}_t^2]] = (1/2)\mathbf{I}_N$), and thus eq. (B.4) implies that $\mathbb{E}(\mathbf{D}_{t,k}) = \mathbf{C}^k$.

ii)Let $\rho_{\max}(\mathbf{C})$ refer to the modulus of the largest eigenvalue of $\mathbf{C}$.

iii) Let $(\Omega, \mathcal{F}, P)$ be a probability space and $L_2(\Omega, \mathcal{F}, P)$ (in short L_2) be the Hilbert space of random variables with finite first and second moments defined on $(\Omega, \mathcal{F}, P)$.

Condition 1 $\rho_{\max}(\mathbf{C}) < 1$.

Taking the conditional expectation of eq. (B.5) with respect to the σ field $\mathcal{F}_{t-k-1}$ yields the following Proposition.

Proposition 2 *The k-step-ahead optimal (in L_2 sense) linear predictor of the N-dimensional AP-HEAVY(1, 1) model is readily seen to be*

$$\mathbb{E}(\boldsymbol{\sigma}_t^{\wedge\boldsymbol{\delta}} | \mathcal{F}_{t-k-1}) = (\mathbf{I}_N - \mathbf{C})^{-1}(\mathbf{I}_N - \mathbf{C}^k)\boldsymbol{\omega} + \mathbf{C}^k\boldsymbol{\sigma}_{t-k}^{\wedge\boldsymbol{\delta}}. \tag{B.7}$$

Under Condition 1 the unconditional mean of $\boldsymbol{\sigma}_t^{\wedge\boldsymbol{\delta}}$, that is $\boldsymbol{\sigma}(\boldsymbol{\delta}) = \mathbb{E}(\boldsymbol{\sigma}_t^{\wedge\boldsymbol{\delta}})$ is equal to the $\lim_{k\to\infty} \mathbb{E}(\boldsymbol{\sigma}_t^{\wedge\boldsymbol{\delta}} | \mathcal{F}_{t-k-1})$, and thus it is given by

$$\boldsymbol{\sigma} = (\mathbf{I}_N - \mathbf{C})^{-1}\boldsymbol{\omega}. \tag{B.8}$$

(where $\mathbf{C}$ has been defined in eq. (B.6)).

Finally, the following Proposition gives the optimal linear predictor of the power transformed observed vector $|\mathbf{r}_t|^{\wedge\boldsymbol{\delta}}$ as well as its first unconditional moment.

Proposition 3 *The k-step-ahead optimal (in L_2 sense) linear predictor of the power transformed observed vector $|\mathbf{r}_t|^{\wedge\boldsymbol{\delta}}$ is given by*

$$\mathbb{E}(|\mathbf{r}_t|^{\wedge\boldsymbol{\delta}} | \mathcal{F}_{t-k-1}) = \mathbf{Z}\mathbb{E}(\boldsymbol{\sigma}_t^{\wedge\boldsymbol{\delta}} | \mathcal{F}_{t-k-1}),$$

($\mathbf{Z}$ has been defined in Definition 1(i), and eq. (B.7) gives $\mathbb{E}(\boldsymbol{\sigma}_t^{\wedge\boldsymbol{\delta}} | \mathcal{F}_{t-k-1})$).
Under Condition 1, the unconditional mean of $|\mathbf{r}_t|^{\wedge\boldsymbol{\delta}}$, that is $\mathbf{r}(\boldsymbol{\delta}) = \mathbb{E}(|\mathbf{r}_t|^{\wedge\boldsymbol{\delta}})$ is equal to $\lim_{k\to\infty} \mathbb{E}(|\mathbf{r}_t|^{\wedge\boldsymbol{\delta}} | \mathcal{F}_{t-k-1})$, and thus it is given by

$$\mathbf{r} = \mathbf{Z}\boldsymbol{\sigma}. \tag{B.9}$$

The proof is trivial. It follows from the definition of $|\mathbf{r}_t|^{\wedge\boldsymbol{\delta}}$ in eq. (B.1) and Proposition 2. Alternatively, we could obtain the optimal linear predictor and the first unconditional moment of $|\mathbf{r}_t|^{\wedge\boldsymbol{\delta}}$ using its weak VARMA$(1,1)$ representation, which is not difficult to show (proof not reported but it is available upon request) and it is given by:

$$[\mathbf{I}_N - L\mathbf{C}_t]\,|\mathbf{r}_t|^{\wedge\boldsymbol{\delta}} = \mathbf{Z}\boldsymbol{\omega} + (\mathbf{I}_N - \mathbf{B}L)\mathbf{v}_t.$$

C Second Moments

Now that we have derived the optimal predictors and the first unconditional moment of the AP-HEAVY system, we will examine its second moment structure.

C.1 Notation

But first, we will introduce some further notation.

Covariances

Let $\boldsymbol{\Gamma}(\ell;\boldsymbol{\delta}) = [\gamma_{ij}(\ell;\boldsymbol{\delta})]$, $\ell \in \mathbb{Z}_{\geq 0}$, be the multidimensional covariance function of $\{\boldsymbol{\sigma}_t^{\wedge\boldsymbol{\delta}}\}$; as usual in what follows we will suppress the subscript $\boldsymbol{\delta}$ for ease of notation, that is we will use $\boldsymbol{\Gamma}(\ell;\boldsymbol{\delta}) = \boldsymbol{\Gamma}(\ell)$. In view of this definition we have:

$$\boldsymbol{\Gamma}(\ell) = \mathbb{E}[(\boldsymbol{\sigma}_{t-\ell}^{\wedge\boldsymbol{\delta}} - \boldsymbol{\sigma})(\boldsymbol{\sigma}_t^{\wedge\boldsymbol{\delta}} - \boldsymbol{\sigma})'] = \boldsymbol{\Sigma}(\ell) - \boldsymbol{\sigma}\boldsymbol{\sigma}', \tag{C.1}$$

where $\boldsymbol{\Sigma}(\ell) = \mathbb{E}(\boldsymbol{\sigma}_{t-\ell}^{\wedge\boldsymbol{\delta}}(\boldsymbol{\sigma}_t^{\wedge\boldsymbol{\delta}})')$. In addition, let the vectorizations of $\boldsymbol{\Sigma}(\ell)$ and $\boldsymbol{\Gamma}(\ell)$ be denoted by $\mathbf{s}(\ell)$ and $\boldsymbol{\gamma}(\ell)$, respectively. Explicit solutions for the $\boldsymbol{\Gamma}(\ell)$ and conditions for its existence will be presented below.

Further, let

$$\mathbf{D} = diag[\sqrt{\gamma_{11}(0)}, \ldots, \sqrt{\gamma_{NN}(0)}],$$

where $\gamma_{ii}(0)$ is the element occupying the i-th diagonal entry of $\boldsymbol{\Gamma}(0)$. To further fix notation, write the ℓ-th-order, for $\ell \geq 1$, autocorrelation matrix of $\boldsymbol{\sigma}_t^{\wedge\boldsymbol{\delta}}$ as

$$\mathbf{R}(\ell) = \mathbf{D}^{-1}\boldsymbol{\Gamma}(\ell)\mathbf{D}^{-1}.$$

Kronecker Products

In what follows we will introduce some additional notation, which involves various Kronecker products. Specifically, let

$$\mathbf{C}^{\otimes 2} = \mathbf{C} \otimes \mathbf{C}, \quad \overline{\mathbf{A}}^{\otimes 2} = \overline{\mathbf{A}} \otimes \overline{\mathbf{A}}, \tag{C.2}$$

where $\mathbf{C}$ and $\overline{\mathbf{A}}$ have been defined in eq. (B.6).

We continue by introducing the following notation.

Notation 1 *Let*

$$\mathbf{Z}^{\otimes 2} = \mathbf{Z} \otimes \mathbf{Z},\ \mathbb{E}\left[\left(|\mathbf{Z}_t|^{\wedge\boldsymbol{\delta}}\right)^{\otimes 2}\right] = \mathbb{E}(|\mathbf{Z}_t|^{\wedge\boldsymbol{\delta}} \otimes |\mathbf{Z}_t|^{\wedge\boldsymbol{\delta}}),$$
$$\widetilde{\mathbf{Z}} = \left[\mathbb{E}\left(|\mathbf{Z}_t|^{\wedge\boldsymbol{\delta}}\right)^{\otimes 2}\right] - \mathbf{Z}^{\otimes 2} = \mathbb{E}\left[\left(|\mathbf{Z}_t|^{\wedge\boldsymbol{\delta}} - \mathbf{Z}\right)^{\otimes 2}\right],$$

be two diagonal matrices of order N^2 ($\mathbf{Z}_t$ and $\mathbf{Z}$ have been defined in eq. (B.1) and Definition 1(i), respectively).

Remark 2 *The element occupying the r-th diagonal entry of $\widetilde{\mathbf{Z}}$, with $r = [(i-1)N + j]$, where $i, j = 1, \ldots, N$, is given by*

$$\mathbb{E}(|e_{it}|^{\delta_i}\,|e_{jt}|^{\delta_j}) - \mathbb{E}(|e_{(it}|^{\delta_i})\mathbb{E}(|e_{jt}|^{\delta_j}).$$

C.2 Covariance Structure

In the following theorem, we will present an explicit formula for $\boldsymbol{\gamma}(0)$. First, let

$$\widetilde{\mathbf{C}} = \mathbf{C}^{\otimes 2} + \overline{\mathbf{A}}^{\otimes 2}\widetilde{\mathbf{Z}} \tag{C.3}$$

(where $\mathbf{C}^{\otimes 2}$ and $\overline{\mathbf{A}}^{\otimes 2}$ are given in eq. (C.2), and $\widetilde{\mathbf{Z}}$ is defined in Notation 1.

Condition 2 $\rho_{\max}(\widetilde{\mathbf{C}}) < 1$.

Theorem 2 *Consider the N-dimensional vector AP-HEAVY $(1,1)$ process. Under Condition 2 the vectorization of $\boldsymbol{\Gamma}(0)$, is given by*

$$\boldsymbol{\gamma}(0) = \left(\mathbf{I}_{N^2} - \widetilde{\mathbf{C}}\right)^{-1}\overline{\mathbf{A}}^{\otimes 2}\widetilde{\mathbf{Z}}\boldsymbol{\sigma}^{\otimes 2}. \tag{C.4}$$

Further, $\boldsymbol{\gamma}(\ell)$, for $\ell \geq 1$, is given by

$$\boldsymbol{\gamma}(\ell) = \left(\mathbf{C}^{\ell} \otimes \mathbf{I}_N\right)\boldsymbol{\gamma}(0). \tag{C.5}$$

Next, let us denote the multidimensional covariance function of $\{|\mathbf{r}_t|^{\wedge\boldsymbol{\delta}}\}$ by $\boldsymbol{\Gamma}_r(\ell) = [\gamma_{ij,r}(\ell)]$.

Theorem 3 *Consider the N-dimensional vector AP-HEAVY $(1,1)$ process. Under Condition 2 the vectorization of $\mathbf{\Gamma}_r(0)$, is given by*

$$\boldsymbol{\gamma}_r(0) \;=\; \left[\mathbb{E}\left[\left(|\mathbf{Z}_t|^{\wedge\boldsymbol{\delta}}\right)^{\otimes 2}\right]\left(\mathbf{I}_{N^2}-\widetilde{\mathbf{C}}\right)^{-1}\overline{\mathbf{A}}^{\otimes 2}+\mathbf{I}_{N^2}\right]\widetilde{\mathbf{Z}}\boldsymbol{\sigma}^{\otimes 2} \tag{C.6}$$

Similarly, $\boldsymbol{\gamma}_r(\ell)$, for $\ell \geq 1$, is given by

$$\boldsymbol{\gamma}_r(\ell) = \mathbf{Z}\boldsymbol{\gamma}_r(0). \tag{C.7}$$

C.3 *S*econd Moments (Proofs)

Next, we will present the proofs of Theorems 2 and 3. But first we present the following lemma that we will use in the proofs below.

Lemma 1 *The vec $\left[\mathbb{E}\left(\mathbf{A}_{t-1}\mathbf{v}_{t-1}\mathbf{v}'_{t-1}\mathbf{A}'_{t-1}\right)\right]$ is given by*

$$vec\left[\mathbb{E}\left(\mathbf{A}_{t-1}\mathbf{v}_{t-1}\mathbf{v}'_{t-1}\mathbf{A}'_{t-1}\right)\right] \;=\; \overline{\mathbf{A}}^{\otimes 2}\widetilde{\mathbf{Z}}\left[\boldsymbol{\gamma}(0)+\boldsymbol{\sigma}^{\otimes 2}\right]. \tag{C.8}$$

Proof. Using the definition of $\mathbf{v}_{t-1}$ in Definition 1(ii) and interchanging the vec and expectation operators, the left hand side of eq. (C.8) takes the form:

$$\mathbb{E}\left\{vec\left[\mathbf{A}_{t-1}\left(|\mathbf{Z}_t|^{\wedge\boldsymbol{\delta}}-\mathbf{Z}\right)\boldsymbol{\sigma}_t^{\wedge\boldsymbol{\delta}}(\boldsymbol{\sigma}_t^{\wedge\boldsymbol{\delta}})'\left(|\mathbf{Z}_t|^{\wedge\boldsymbol{\delta}}-\mathbf{Z}\right)'\mathbf{A}'_{t-1}\right]\right\}.$$

Using the rules of the vec operator (see, for example, Lütkepohl, 1996, Section 7.2) and, under Condition 2, applying the expectation operator, in view of eq. (C.1) the above expression yields

$$vec\left[\mathbb{E}\left(\mathbf{A}_{t-1}\mathbf{v}_{t-1}\mathbf{v}'_{t-1}\mathbf{A}'_{t-1}\right)\right] \;=\; \mathbb{E}\left(\mathbf{A}_{t-1}^{\otimes 2}\right)\mathbb{E}\left(|\mathbf{Z}_t|^{\wedge\boldsymbol{\delta}}-\mathbf{Z}\right)^{\otimes 2}(\boldsymbol{\gamma}(0)+\boldsymbol{\sigma}^{\otimes 2}). \tag{C.9}$$

Since $\mathbb{E}\left(\mathbf{A}_t^{\otimes 2}\right)=\overline{\mathbf{A}}^{\otimes 2}$ and in view of Notation 1, it follows that the right hand-side of eq. (C.9) equals the right hand-side of eq. (C.8) as required. ■

Proof. (**of Theorem 2**) Rewrite the weak VARMA representation, eq. (B.3), as

$$\boldsymbol{\sigma}_t^{\wedge\boldsymbol{\delta}} = \boldsymbol{\omega} + \mathbf{C}_{t-1}\boldsymbol{\sigma}_{t-1}^{\wedge\boldsymbol{\delta}} + \mathbf{A}_{t-1}\mathbf{v}_{t-1}.$$

Using $\boldsymbol{\omega} = (\mathbf{I}_N - \mathbf{C})\boldsymbol{\sigma}$ (see eq. (B.8)) the above equation can be expressed in terms of deviations from the mean:

$$\boldsymbol{\sigma}_t^{\wedge\boldsymbol{\delta}} - \boldsymbol{\sigma} = (\mathbf{C_{t-1}} - \mathbf{C})\boldsymbol{\sigma} + \mathbf{C}_{t-1}(\boldsymbol{\sigma}_{t-1}^{\wedge\boldsymbol{\delta}} - \boldsymbol{\sigma}) + \mathbf{A}_{t-1}\mathbf{v}_{t-1}. \tag{C.10}$$

Taking the transpose on both sides of eq. (C.10) yields

$$(\boldsymbol{\sigma}_t^{\wedge\boldsymbol{\delta}} - \boldsymbol{\sigma})' = \boldsymbol{\sigma}'(\mathbf{C_{t-1}} - \mathbf{C})' + (\boldsymbol{\sigma}_{t-1}^{\wedge\boldsymbol{\delta}} - \boldsymbol{\sigma})'\mathbf{C}'_{t-1} + \mathbf{v}'_{t-1}\mathbf{A}'_{t-1}. \tag{C.11}$$

Right-multiplying eq. (C.10) by eq. (C.11) and, under Condition 2, taking expectations on both sides, yields (in view of eq. (C.1) and ignoring zero terms):

$$\boldsymbol{\Gamma}(0) = \mathbb{E}\left[\mathbf{C}_{t-1}(\boldsymbol{\sigma}_{t-1}^{\wedge\boldsymbol{\delta}} - \boldsymbol{\sigma})(\boldsymbol{\sigma}_{t-1}^{\wedge\boldsymbol{\delta}} - \boldsymbol{\sigma})'\mathbf{C}_{t-1}'\right] + \mathbb{E}\left(\mathbf{A}_{t-1}\mathbf{v}_{t-1}\mathbf{v}_{t-1}'\mathbf{A}_{t-1}'\right). \tag{C.12}$$

Applying the vec operator to both sides of eq. (C.12) yields

$$\boldsymbol{\gamma}(0) = \mathbb{E}\left(\mathbf{C}_t^{\otimes 2}\right)\boldsymbol{\gamma}(0) + vec\left[\mathbb{E}\left(\mathbf{A}_{t-1}\mathbf{v}_{t-1}\mathbf{v}_{t-1}'\mathbf{A}_{t-1}'\right)\right].$$

In view of Lemma 1 and the fact that $\mathbb{E}\left(\mathbf{C}_t^{\otimes 2}\right) = \mathbf{C}^{\otimes 2}$, we have

$$\boldsymbol{\gamma}(0) = \mathbf{C}^{\otimes 2}\boldsymbol{\gamma}(0) + \overline{\mathbf{A}}^{\otimes 2}\widetilde{\mathbf{Z}}\left[\boldsymbol{\gamma}(0) + \boldsymbol{\sigma}^{\otimes 2}\right].$$

Solving the above equation for $\boldsymbol{\gamma}(0)$ gives

$$\boldsymbol{\gamma}(0) = \left(\mathbf{I}_{N^2} - \widetilde{\mathbf{C}}\right)^{-1}\overline{\mathbf{A}}^{\otimes 2}\widetilde{\mathbf{Z}}\boldsymbol{\sigma}^{\otimes 2}$$

which completes the proof of eq. (C.4).

Next, rewrite the general solution in eq. (B.5) as

$$(\boldsymbol{\sigma}_t^{\wedge\boldsymbol{\delta}})' = \sum_{r=1}^{\ell}(\boldsymbol{\omega}' + \mathbf{v}_{t-r}'\mathbf{A}_{t-r}')\mathbf{D}_{t,r-1}' + (\boldsymbol{\sigma}_{\mathbf{t}-\ell}^{\wedge\boldsymbol{\delta}})'\mathbf{D}_{t,\ell}'.$$

Left-multiplying the above equation by $\boldsymbol{\sigma}_{t-\ell}^{\wedge\boldsymbol{\delta}}$, taking expectations on both sides under Condition 2, and using $\mathbb{E}(\mathbf{D}_{t,\ell}) = \mathbf{C}^{\ell}$, see the text next to eq. (B.6), yields (in view of eq. (C.1) and ignoring zero terms):

$$\boldsymbol{\Sigma}(\ell) = \boldsymbol{\sigma}\boldsymbol{\omega}'[(\mathbf{I} - \mathbf{C})^{-1}]'(\mathbf{I} - \mathbf{C}^{\ell})' + \boldsymbol{\Sigma}(0)(\mathbf{C}^{\ell})'.$$

On account of $\boldsymbol{\omega} = (\mathbf{I} - \mathbf{C})\boldsymbol{\sigma}$, it follows that

$$\boldsymbol{\Gamma}(\ell) = \boldsymbol{\Gamma}(0)(\mathbf{C}^{\ell})'.$$

Applying the vec operator to both side of the above equation yields eq. (C.5) as claimed. ■

Proof. (**of Theorem 3**) Rewrite $|\mathbf{r}_t|^{\wedge\boldsymbol{\delta}}$ in terms of deviations from the mean (see eqs. (B.1) and (B.9)):

$$|\mathbf{r}_t|^{\wedge\boldsymbol{\delta}} - \mathbf{r} = |\mathbf{Z}_t|^{\wedge\boldsymbol{\delta}}(\boldsymbol{\sigma}_t^{\wedge\boldsymbol{\delta}} - \boldsymbol{\sigma}) + \left(|\mathbf{Z}_t|^{\wedge\boldsymbol{\delta}} - \mathbf{Z}\right)\boldsymbol{\sigma} \text{ or}$$
$$\left(|\mathbf{r}_t|^{\wedge\boldsymbol{\delta}} - \mathbf{r}\right)' = (\boldsymbol{\sigma}_t^{\wedge\boldsymbol{\delta}} - \boldsymbol{\sigma})'\left(|\mathbf{Z}_t|^{\wedge\boldsymbol{\delta}}\right)' + \boldsymbol{\sigma}'\left(|\mathbf{Z}_t|^{\wedge\boldsymbol{\delta}} - \mathbf{Z}\right)'.$$

Multiplying $|\mathbf{r}_t|^{\wedge\boldsymbol{\delta}} - \mathbf{r}$ by its transpose, using the above expressions, taking expectations on both sides, and ignoring zero terms, it follows that the vectorization of $\boldsymbol{\Gamma}_r(0)$ is given by

$$\boldsymbol{\gamma}_r(0) = \mathbb{E}\left[\left(|\mathbf{Z}_t|^{\wedge\boldsymbol{\delta}}\right)^{\otimes 2}\right]\boldsymbol{\gamma}(0) + \widetilde{\mathbf{Z}}\boldsymbol{\sigma}^{\otimes 2}.$$

Applying eq. (C.4) to the above expression of $\boldsymbol{\gamma}_r(0)$, eq. (C.6) follows (the proof of eq. (C.7) is similar to the proof of eq. (C.6) and, thus it is omitted) and the proof is complete. ■

D The Bovespa Index

D.1 Optimal Predictiors

We provide a comparison between the benchmark HEAVY system and the more general AP specification. Their difference is captured by the matrix $\mathbf{C}$ (see eq. (B.6)). We will examine the bivariate case, which is when $N = 2$. For the more general DAP specification, $\mathbf{C}$ is a full matrix with: i) diagonal elements given by $\beta_i + (\alpha_{ii} + \gamma_{ii}/2)z_i$, $i = r, R$, where $z_i = \mathbb{E}(|e_{it}|^{\delta_i}$, and ii) off-diagonal elements given by $(\alpha_{ij} + \gamma_{ij})z_j$, $i, j = r, R$, for $i \neq j$. For the benchmark model, since $\gamma_{ij} = 0$, $z_i = 1$, for all $i, j = r, R$, and $\alpha_{Ri} = 0$, $\mathbf{C}$ is restricted to being an upper diagonal matrix. That is, we have

$$
\text{DAP Specification:} \qquad \mathbf{C} = \begin{bmatrix} \beta_r + (\alpha_{rr} + \gamma_{rr}/2)z_r & (\alpha_{rR} + \gamma_{rR}/2)z_R \\ (\alpha_{Rr} + \gamma_{Rr}/2)z_r & \beta_R + (\alpha_{RR} + \gamma_{RR}/2)z_R \end{bmatrix}
$$

$$
\text{Benchmark HEAVY} \quad : \quad \mathbf{C} = \begin{bmatrix} \beta_r & \alpha_{rR} \\ 0 & \beta_R + \alpha_{RR} \end{bmatrix}.
$$

Figure D.1 presents the comparison of the benchmark and DAP-HEAVY models' forecasting performance (see also Section 6 in the main body of the paper). We apply the optimal predictor $|\mathbf{r}_t|^{\wedge\boldsymbol{\delta}}$ (under Proposition 3) on Brazil's Bovespa index returns and realized variance data and calculate 50-step ahead forecasts. The more general specification produces forecasts significantly closer to the actual values for both returns (Fig. D.1, a & b) and realized measure (Fig. D.1, c & d). Most importantly, its forecasts are more accurate in peaks of returns and realized variance actual values. The benchmark model remains behind our proposed asymmetric power extension in predicting low- and high-frequency volatility indicators. It produces, mostly, lower volatility forecasts (dotted lines) in comparison with the DAP (dashed lines) and actual (solid lines) values. Therefore, our main contribution, that is the asymmetric power extension, provides a significant improvement to the HEAVY system of Shephard and Sheppard (2010).

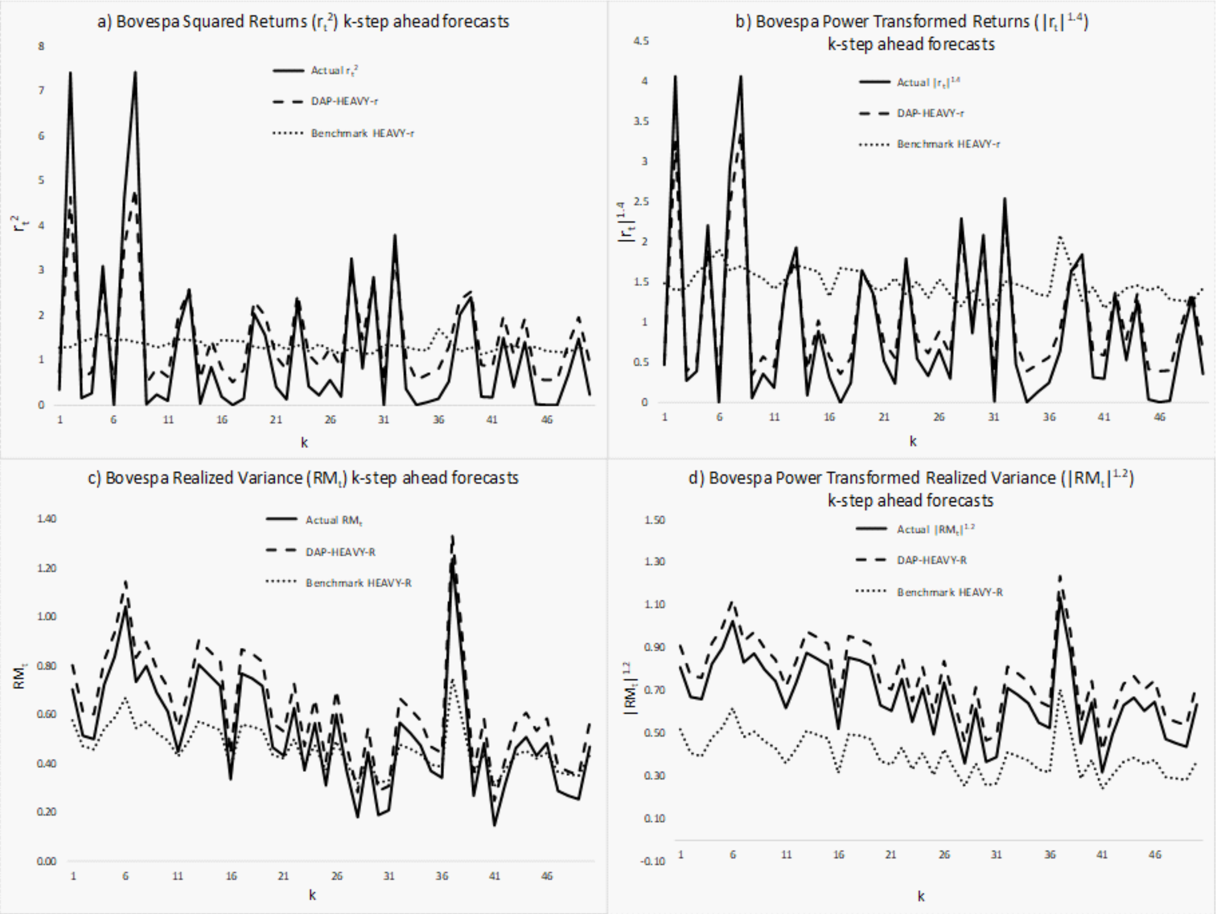

Figure D.1. Bovespa Returns and Realized Variance k-step ahead forecasts

D.2 Sample Autocorrelations

Next, we examine the sample autocorrelations of the power transformed absolute returns $|r_t|^{\delta_r}$ and signed square rooted realized variance $|SSR\_RM_t|^{\delta_R}$ for various values of δ_i. Figures D.2 and D.3 show the autocorrelograms of Brazil's Bovespa index from lag 1 to 120 for $\delta_r = 1.4, 1.7, 2.0$ and $\delta_R = 1.2, 1.6, 2.0$ (similar autocorrelograms for the other four indices available upon request). The sample autocorrelations for $|r_t|^{1.4}$ are greater than the sample autocorrelations of $|r_t|^{\delta_r}$ for $\delta_r = 1.7, 2.0$ at every lag up to at least 120 lags. In other words, the most interesting finding from the autocorrelogram is that $|r_t|^{\delta_r}$ has the strongest and slowest decaying autocorrelation when $\delta_r = 1.4$. Similarly, for the realized measure, the power with the strongest autocorrelation function is $\delta_R = 1.2$. Furthermore, Figures D.4 and D.5 present the sample autocorrelations of $|r_t|^{\delta_r}$ and $|SSR\_RM_t|^{\delta_R}$ as a function of δ_i for lags $1, 12, 36, 72$ and 96. For example, for lag 12, the highest autocorrelation values of power transformed absolute returns and signed square rooted realized variance are calculated closer to the power of 1.5 and 1.0, respectively.

These figures explain our motivation to extend the benchmark HEAVY through the APARCH framework of Ding et al. (1993) and confirm the power choice of our econometric models, which is $\delta_r = 1.4$ for returns and $\delta_R = 1.2$ for the realized measure (see Section 5 in the main body of the paper).

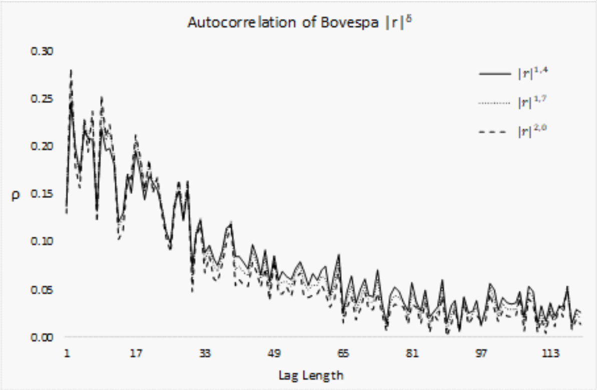

Figure D.2. Autocorrelation of Bovespa $|r_t|^{\delta_r}$ for $\delta_r = 1.4, 1.7, 2.0$

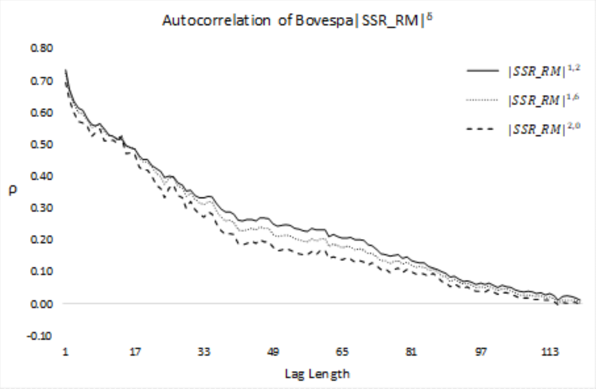

Figure D.3. Autocorrelation of Bovespa $|SSR\_RM_t|^{\delta_R}$ for $\delta_R = 1.2, 1.6, 2.0$

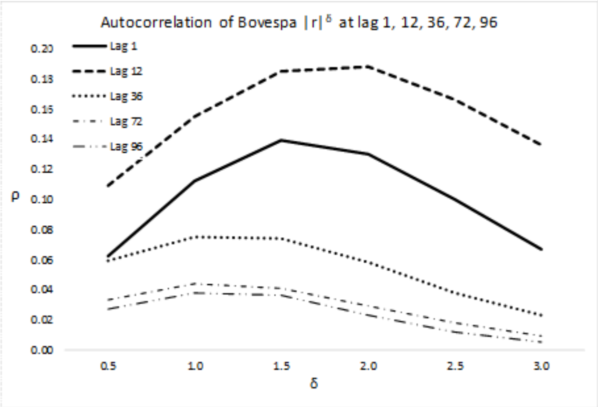

Figure D.4. Autocorrelation of Bovespa $|r_t|^{\delta_r}$ at lags $1, 12, 36, 72, 96$

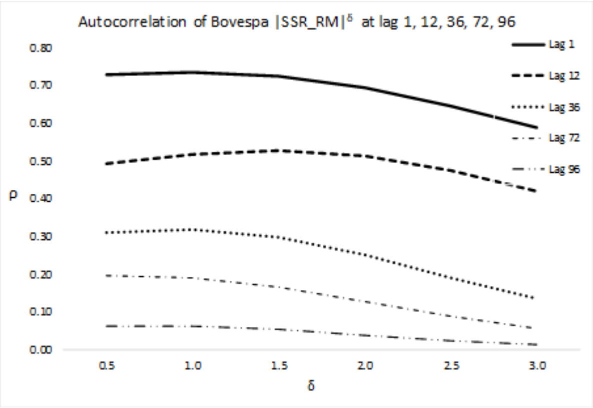

Figure D.5. Autocorrelation of Bovespa $|SSR\_RM_t|^{\delta_R}$ at lags $1, 12, 36, 72, 96$

E Stock Index and Residuals Graphs

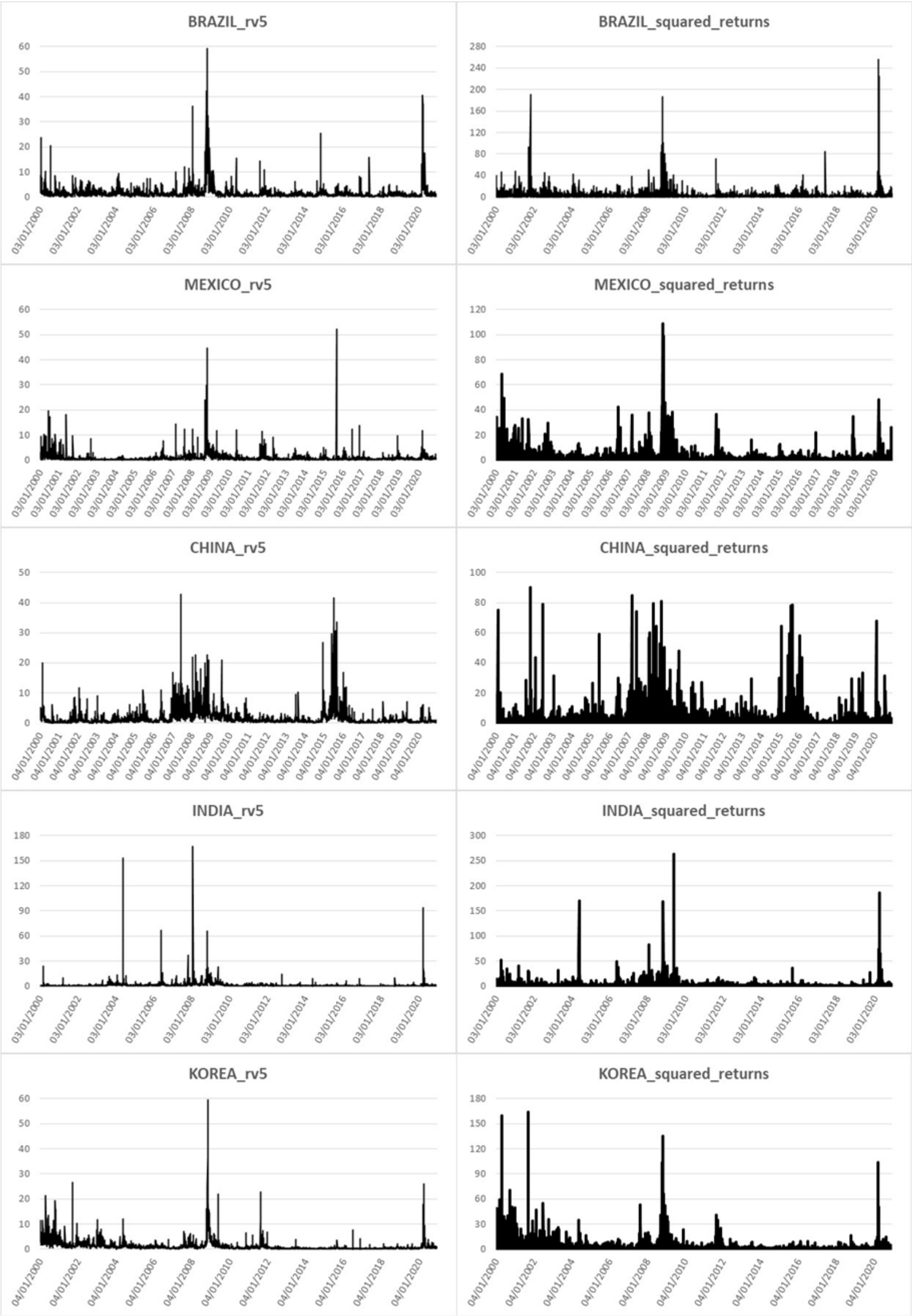

Figure E.1. Stock index Realized Variance and Squared Returns

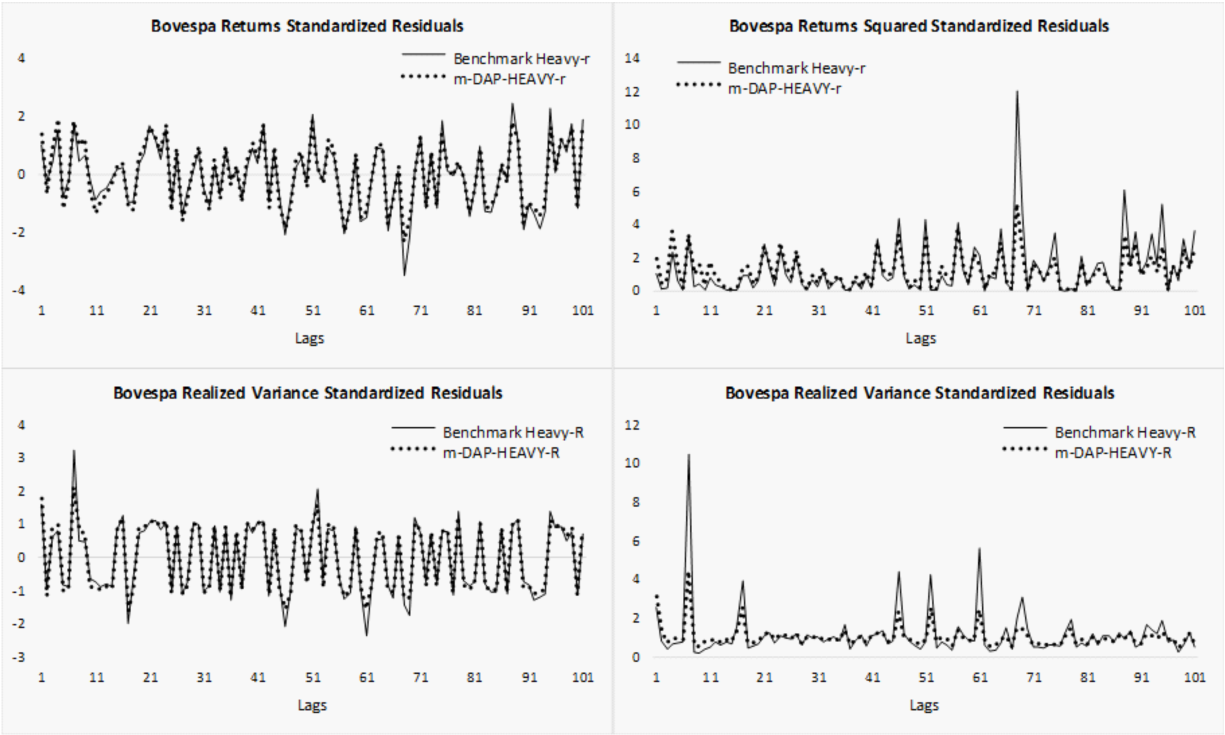

Figure E.2. Brazil's Bovespa Residuals (Benchmark HEAVY and m-DAP-HEAVY models)

References

[1] Lütkepohl, H., 1996. Handbook of Matrices (Vol. 1). Chichester: Wiley.
